# Gestational PFAS exposure and newborn size: The modifying effect of cord blood fatty acids

**DOI:** 10.1016/j.ese.2024.100476

**Published:** 2024-08-03

**Authors:** Chang Gao, Lin Luo, Yijun Fan, Liyan Guo, Lijuan Guo, Lin Tao, Fangbiao Tao, De-Xiang Xu, Robert A. Gibson, Maria Makrides, Hua Wang, Yichao Huang

**Affiliations:** a Department of Toxicology, School of Public Health, Anhui Medical University, Hefei, 230031, China; b Key Laboratory of Environmental Toxicology of Anhui Higher Education Institutes, Anhui Medical University, Hefei, 230031, China; c Center for Big Data and Population Health of IHM, Anhui Medical University, Hefei, 230031, China; d MOE Key Laboratory of Population Health Across Life Cycle, Anhui Medical University, Hefei, 230031, China; e Department of Gynecology and Obstetrics, The Second Affiliated Hospital of Anhui Medical University, Hefei, 230601, China; f Department of Maternal & Child and Adolescent Health, School of Public Health, Anhui Medical University, Hefei, 230031, China; g SAHMRI Women and Kids, South Australian Health and Medical Research Institute, North Adelaide, 5000, South Australia, Australia; h Clinical Research Center, Suzhou Hospital of Anhui Medical University, Suzhou, 234099, China

**Keywords:** Per- and polyfluoroalkyl substance, Gestational exposure, Fetal nutrition, Fatty acid status, Newborn size

## Abstract

Per- and polyfluoroalkyl substances (PFASs) can disrupt lipid metabolism, and changes in cord blood fatty acid composition have been observed in small newborns. Emerging evidence suggests that exposure to PFASs during pregnancy is linked to decreased newborn size, although the evidence is not consistent. The modifying effect of fatty acids on the associations of gestational PFAS exposure with newborn size is still unknown. Here we show that the nutritional status of the fetus, as indicated by the level of fatty acids in the cord blood, mitigates the adverse effects of gestational PFAS exposure on the size of the newborn. Our study confirms the adverse developmental effects of PFASs and identifies emerging short-chain PFASs as the primary drivers of reduced newborn size, despite their lower exposure burden compared to legacy PFASs. Additionally, we find the protective role of cord blood fatty acids, suggesting potential strategies for mitigating the detrimental effects of emerging environmental exposures on human health. Our findings provide new evidence of the potential toxicity of emerging PFASs and call for further toxicity evaluations of these pollutants for regulatory purposes. Future studies should consider the complex interaction between exposure and nutrition within the human body, particularly during the first thousand days of life, to promote lifelong health.

## Highlights

• Eleven legacy and emerging PFASs were detected in nearly all participants.
• Gestational exposure to PFASs mixture were related to reduced newborn size.
• The effect was mainly associated with emerging PFASs containing carboxylic group.
• Cord blood SFA and MUFA moderated the effect of PFASs on newborn size.

## Graphical abstract

**Figure:**
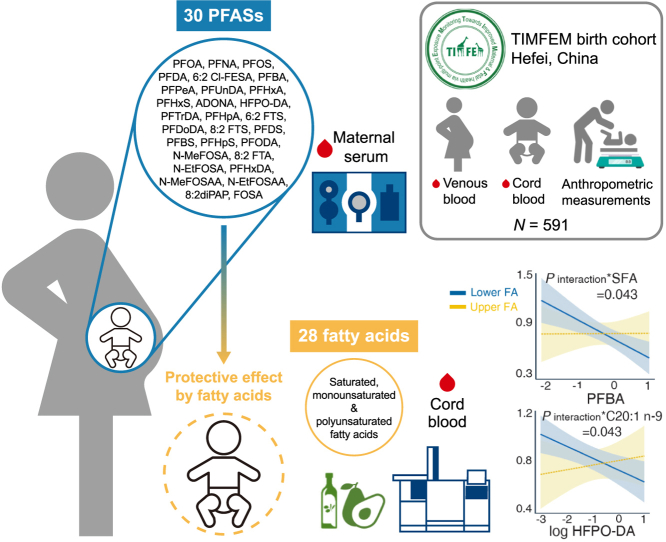

## 1 Introduction

Per- and polyfluoroalkyl substances (PFASs) are a class of chemicals with high stability and durability, which repel oil, water, and dirt [1]. Their application in industrial and commercial uses (e.g., nonstick pans, fabrics, and cleansing products) has steadily increased since the 1950s [2,3]. Due to their widespread use and environmental persistency, PFASs have been detected in soil [4], water [5], and air [6] across the globe, despite some regional variations, and identified in human bodily fluids and tissues across different age groups [7]. The first thousand days of life are the cornerstone for the lifelong health of individuals, making them a matter of particular concern. Although the results are inconsistent, there has been epidemiological evidence supporting an association between gestational PFAS exposure and adverse birth outcomes (e.g., low birth weight and preterm birth) [[8], [9], [10], [11], [12]].

The current evidence regarding the relationship between gestational PFAS exposure and newborn birth size has two major shortfalls. First, most studies focused on a few selected PFASs, in addition to perfluorooctanoic acid (PFOA) and perfluorooctanesulfonic acid (PFOS) [[13], [14], [15], [16], [17]]. Considering their health and environmental toxicity, these legacy PFASs have been banned from global production. With their gradual phase-out, emerging PFASs with similar chemical properties but shorter chain lengths have been developed as replacements. However, the enhanced hydrophilicity associated with a reduced chain length has made emerging PFASs even more difficult to eliminate from water bodies [18], meaning they pose greater harm to humans than the legacy PFASs they have replaced. In an additional limitation, certain recent reports on the topic were based on dated samples [[19], [20], [21]], where the measured values were unlikely to reflect real exposure burdens today. Hence, they could not provide timely information regarding the health risks of newly emerging PFASs.

The role of PFAS exposure in disturbing lipid metabolism by inducing peroxisome proliferator-activated receptor-α (PPAR-α) activation [22] or cytochrome P450 gene expression [23] has long been established. Gestational PFAS exposure has been related to alterations in maternal lipid and fatty acid (FA) metabolism, especially perturbed monounsaturated fatty acid (MUFA) homeostasis, which has been linked to a risk of small-for-gestational-age (SGA) fetuses [24]. In addition, an altered cord blood FA profile featuring a reduced level of long-chain saturated fatty acids (SFAs) has been noted in SGA neonates [25]. Against that research background, we aimed to determine whether the cord blood FA could attenuate the adverse effect of gestational PFAS exposure on the newborn size.

## 2 Materials and methods

### 2.1 Study population and sample collection

Participants included in this study were part of an ongoing prospective birth cohort being studied in Hefei, Anhui Province, China, under the Towards Improved Maternal & Fetal health via multi-point Exposure Monitoring (TIMFEM) study. From December 2021 onwards, women aged between 18 and 45 at the early stage of singleton pregnancy (6–12 weeks), seeking antenatal check-ups and planning to deliver at the Second Affiliated Hospital of Anhui Medical University, were approached for recruitment, and those who provided written consent were enrolled.

The exclusion criteria for the TIMFEM study were as follows: women who received assisted reproductive technology for their pregnancy had a major chronic disease or infectious disease, were unwilling to participate, or planned to attend antenatal visits and/or deliver outside the designated hospital. The study protocol was approved by the Ethics Committee of the Second Affiliated Hospital of Anhui Medical University (YX2021-091F1).

For the current study, 591 mothers were included, all delivering a live newborn infant. We obtained matched maternal serum collected before delivery, cord blood, and a completed prenatal questionnaire for each participant. The maternal and cord venous blood samples were centrifuged, and serum was separated and stored at −80 °C before processing and analysis.

### 2.2 Exposure assessment

We measured the following 30 PFASs from maternal serum: PFOA, perfluorononanoic acid (PFNA), PFOS, perfluorodecanoic acid (PFDA), 6:2 chlorinated polyfluorinated ether sulfonate (6:2 Cl-PFESA), perfluorobutanoic acid (PFBA), perfluoropentanoic acid (PFPeA), perfluoroundecanoic acid (PFUnDA), perfluorohexanoic acid (PFHxA), perfluorohexanesulfonic acid (PFHxS), dodecafluoro-3H-4,8dioxanonanoate (ADONA), hexafluoropropylene oxide dimer acid (HFPO-DA), perfluorotridecanoic acid (PFTrDA), perfluoroheptanoic acid (PFHpA), 6:2 fluorotelomer sulfonate (6:2 FTS), perfluorododecanoic acid (PFDoDA), 8:2 fluorotelomer sulfonate (8:2 FTS), perfluorodecanesulfonic acid (PFDS), perfluorobutanesulfonic acid (PFBS), perfluoroheptanesulfonic acid (PFHpS), perfluorooctadecanoic acid (PFODA), perfluorotetradecanoic acid (PFTeDA), n-methyl-perfluorooctane sulfonamide (N-MeFOSA), 8:2 fluorotelomer acid (8:2 FTA), n-ethylperfluorooctane sulfonamide (N-EtFOSA), perfluorohexadecanoic acid (PFHxDA), n-methylperfluorooctane sulfonamidoacetates (N-MeFOSAA), n-ethylperfluorooctane sulfonamidoacetates (N-EtFOSAA), 8:2 polyfluoroalkyl phosphate diester (8:2 diPAP), and perfluorooctane sulfonamide (FOSA). A full list of those including their chemical abstract service numbers is available in Table S1 (Supplementary Materials)**.**

After defrosting at an ambient temperature, the serum samples were first vortexed, and PFASs were extracted from 50 μL of serum based on our previously reported method [26]. In brief, 20 μL of the internal standard mixture (PFDA-M_2_, PFDoDA-M_2_, PFHxA-M_2_, PFOA-M_4_, PFOS-M_4_, Wellington Laboratories, Guelph, Canada) at a concentration of 0.01 mg L^−1^ was added to the serum sample, followed by the addition of 30 μL of acetonitrile and water (v/v = 1/9). Extraction was then performed with 800 μL of acetonitrile and methanol (v/v = 1/1). After sonication for 10 min, the mixture was left at −20 °C for 2 h to allow protein precipitation. The mixture was then centrifuged at 18,000 *g* for 10 min, and the supernatant was collected and dried under nitrogen flow. The residue was reconstituted with 200 μL methanol and water (v/v = 1/3) and centrifuged in the conditions already described, and the supernatant was separated and used for analysis. All above-mentioned solvents were of high-performance liquid chromatography (HPLC) grade (Fischer Scientific, Hanover Park, IL, USA).

Ultrahigh-performance liquid chromatography coupled with a QTRAP triple quadrupole mass spectrometer (Sciex 5500+, Framingham, MA, USA) and a C18 column (50 mm × 2.1 mm × 1.8 μm, Zorbax Eclipse Plus, Agilent, China) was used for PFASs’ separation and measurement. The mobile phase consisted of 2 mmol L^−1^ of ammonium acetate (phase A) and methanol (phase B) at a flow rate of 0.2 mL min^−1^. A signal/noise ratio of 10 was used to represent the limit of quantification (LOQ), which ranged between 0.002 and 0.098 ng mL^−1^ for all 30 PFASs measured. In further statistical analysis, the LOQ divided by the square root of 2 replaced all PFASs measured below the LOQ.

Routine laboratory quality assurance and quality control were performed with procedural blanks and analyte recoveries from blank and matrix spiking tests. A procedural blank was used for every batch of ten samples, and only trace amounts (average concentration range 0.01–0.04 ng mL^−1^) were detected for PFBA, PFPeA, PFHxA, PFOA, PFNA, and PFOS. Using the same methodology described above, target analytes and surrogate standards were spiked in 50 μL of pooled maternal serum, and the recovery rate was calculated after subtraction of the background levels. Recovery rates for most PFASs were between 45.6% and 117.5% for blank spiking and between 43.4% and 111.6% for matrix spiking (Supplementary Material Table S2).

### 2.3 Fatty acid analysis

We adopted the recommended practice regarding FA analysis and reporting in this study [27]. A total of twenty-eight FAs were measured, including twelve saturated, six monounsaturated, eight polyunsaturated, and two *trans*-fatty acids. Defrosted cord serum samples were vortexed, and 50 μL of each sample was transferred into a glass vial, to which 20 μL of internal standard (heneicosanoic acid, C21:0, 1 mg mL^−1^) was spiked. FAs of all lipid origins were transmethylated into fatty acid methyl esters (FAMEs) in 2 mL of methanol containing 1% sulfuric acid. The transmethylation process took place at 70 °C for 3 h. After completion of the reaction and cooling to an ambient temperature, 250 μL of water and 600 μL of heptane were added to the mixture and vortexed vigorously to allow for extraction and separation. The upper layer was then collected for gas chromatography–mass spectrometry (GC–MS) analysis according to our previously reported method [28,29]. FAME separation was achieved by using an Agilent 8890 GC coupled with a J&W DB-23 column (60 m × 250 μm × 0.25 μm) and an Agilent 5977B MSD detector (Agilent Technologies, California, USA). A mixture of commercialized standards (463 Nu-Chek Prep Inc, Minnesota, USA) was used for individual FAME identification. All FAs were reported as the absolute concentrations (μg mL^−1^).

### 2.4 Newborn birth size

Newborn weight (WZ), length (LZ), and weight-for-length ratio (WLRZ) *z*-scores were calculated based on INTERGROWTH-21st for gestational age and sex-specified standards [30]. Values greater than the absolute value of 5 were considered biologically implausible values (BIVs) and excluded from the analysis.

### 2.5 Covariates

Covariates were chosen based on previous reports or biological considerations, including maternal age (continuous), maternal birth weight (<2500 g; ≥2500, <4000 g; ≥4000 g), maternal pre-pregnancy body mass index (BMI, continuous), gestational weight gain (GWG, continuous), sex (dichotomous), and gestational age of the newborn at birth (continuous). The maternal pre-pregnancy BMI was derived from the self-reported weight and height, and newborn birth characteristics were sourced from medical records.

### 2.6 Statistical analyses

Due to non-normal distribution, all data were presented as median (interquartile range) for continuous variables. Categorical variables were presented as number (percentage). All analyses were restricted to PFASs with a detection frequency of above 60% in the current population.

PFASs and FAs were natural logarithms transformed to normalize the distribution before inclusion in any statistical models. We first assessed the potential linear or non-linear relationship between maternal PFAS exposure and newborn size parameters using multivariate linear regression or the restricted cubic spline model. Then, the mixed effect of PFASs was examined with quantile-based g-computation (QGC) and Bayesian kernel machine regression (BKMR) models. QGC modeling delineates the causal mixed effect on the outcome by simultaneously increasing the concentrations of all joint exposures by one quantile [31]. The contribution (weight) of an individual exposure to the mixed effect can either take a positive or negative direction, and the total weight of each side is one. QGC modeling in the current study was set up with four quantiles and conducted with 20,000 bootstraps. BKMR is another mixed modeling method that allows for flexibility and non-linearities [32], and it was performed with 30,000 iteration cycles in the current study. Plus, the study population was divided based on the cord blood's total FA concentration to further elucidate the role of cord blood FAs in the relationship between PFASs and the newborn size. Those of the lower two tertiles were considered to have a lower FA status, and those of the highest tertile were referred to as having an upper FA status. The mixed effect analyses were performed in the total population and by FA status. In addition, the mixed effect of PFASs on the total and individual cord blood FAs was also determined using the QGC model. Eventually, multivariable linear regression or the restricted cubic spline model was applied to identify a potential effect modification by FAs using the interaction term between the FA status and PFASs. Only PFASs were found to have significant associations with the outcome in the first step of the analysis, and FAs significantly affected by PFASs' mixture were tested in the effect modification analyses. All statistical analyses were performed with R (version 4.3.1; Vienna, Austria), and a two-sided *P*-value ≤0.05 was considered statistically significant.

## 3 Results

### 3.1 Description of the population

Among the 591 mother–infant dyads included in this study, one pair was excluded due to a BIV for LZ, leaving 590 pairs for further analysis, for whom the demographic characteristics are shown in Table 1. The median age of mothers involved in this analysis was 30 (28–32) years, and half of these mothers were primiparous. Nearly 74% of these participating mothers were of normal weight, with a median pre-pregnancy BMI of 21.6 (19.9–23.7) kg m^−2^, and the mothers had a GWG of 15 (11.5–18) kg. Most mothers had completed college (33.9%) or a bachelor's degree or equivalent (37.1%). Over half (55.1%) of the infants in this cohort were male, and the median gestational age at birth was 39 weeks. The median birth weight and length were 3490 (3182–3750) g and 50 (49–51) cm, respectively.

**Table 1:** Demographic characteristics of mother-infant dyads included in the present study.

Characteristics	Whole population
	(*n* = 590)
***Maternal characteristics***	
Maternal age, years, median (IQR)	30 (28, 32)
Pre-pregnancy BMI, median (IQR)	21.6 (19.9, 23.7)
Pre-pregnancy BMI category^a^, *n* (%)	
Underweight	65 (11.0)
Normal weight	436 (73.9)
Overweight	75 (12.7)
Obese	14 (2.4)
GWG, median (IQR)	15 (11.5, 18.0)
Adequacy of GWG^b^, *n* (%)	
Inadequate	116 (19.7)
Adequate	256 (43.4)
Excessive	218 (36.9)
Primiparous, median, *n* (%)	298 (50.5)
Maternal birth weight category, *n* (%)	
<2500 g	23 (3.9)
≥2500, <4000 g	484 (82.0)
≥4000 g	34 (5.8)
Educational level, *n* (%)	
Secondary school or below	135 (22.9)
College	200 (33.9)
Undergraduate	219 (37.1)
Postgraduate	32 (5.4)
***Infant characteristics***	
Sex, Male, *n* (%)	325 (55.1)
Gestational age (weeks), median (IQR)	39 (38.0, 39.8)
Weight at birth (g), median (IQR)	3490 (3,182, 3750)
Weight *z*-score^c^, median (IQR)	0.8 (0.2, 1.4)
Length at birth (cm), median (IQR)	50 (49, 51)
Length *z*-score^c^, median (IQR)	0.9 (0.2, 1.4)
Weight-for-Length Ratio, median (IQR)	6.9 (6.4, 7.4)
Weight-for-Length *z*-score^c^, median (IQR)	0.5 (−0.3, 1.1)

Data are expressed as median (interquartile range, IQR) or *n* (%).

Abbreviations: BMI: body mass index; GWG: gestational weight gain.

a Definition is based on the following standards: underweight (<18.5 kg m^−2^); normal weight (≥18.5, <25 kg m^−2^); overweight (≥25, <30 kg m^−2^) and obese (≥30 kg m^−2^).

b Adopted from the Institute of Medicine (IOM) Weight Gain Recommendation for Pregnancy (2009), the adequate range of gestational weight gain for underweight, normal weight, overweight, and obese mothers before pregnancy is 12.5–18, 11.5–16, 7–11.5, and 5–9 kg (inclusive), respectively. Weight gain below or above the recommended range was considered inadequate or excessive, respectively.

c Derived from INTERGROWTH-21st standard.

### 3.2 Gestational PFAS exposure and newborn size parameters in a single analyte model

The total PFAS concentration of all 30 PFASs was 19.2 (14.9–25.4) ng mL^−1^ (Supplementary Material Table S2). Sixteen of the thirty measured PFASs were detected in more than 60% of the samples: PFOA, PFOS, PFNA, PFDA, 6:2 Cl-PFESA, PFBA, PFPeA, PFUnDA, PFHxA, PFHxS, ADONA, HFPO-DA, PFTrDA, PFHpA, 6:2 FTS, and PFDoDA. Moderate associations (∼0.5) were observed among 6:2 Cl-PFESA, PFOS, PFDA, PFNA, PFTrDA, and PFUnDA; the rest were largely unrelated (Supplementary Material Fig. S1**)**.

**Fig. 1:**
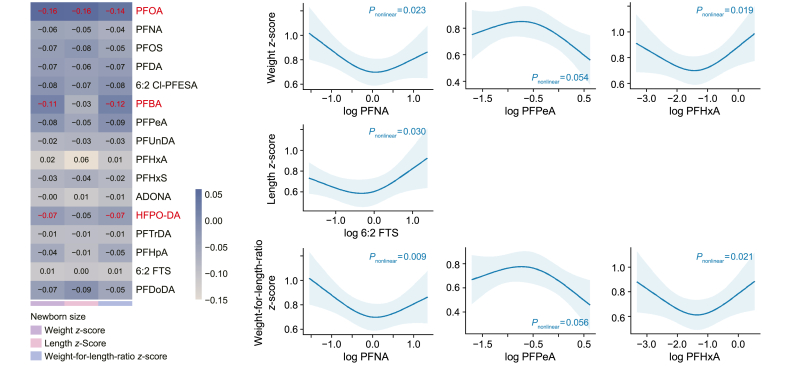
Associations between individual PFAS exposure and newborn size parameters, including weight *z*-score, length *z*-score, and weight-for-length-ratio *z*-score, as determined by linear regression (left) and restricted cubic spline regression (right). Results were adjusted for maternal age, birthweight category (<2500 g; ≥2500, <4000 g; ≥4000 g), maternal pre-pregnancy body mass index, gestational weight gain, sex of newborn, and gestational age at birth. Red text highlight refers to statistically significant associations identified. Please refer to Table S3 (Supplementary Materials) in the supplementary material for detailed information regarding estimates, 95% CI and *P*-values for both linear and non-linear associations.

Associations between individual PFAS exposure and newborn size parameters are visualized in Fig. 1. The per ln-unit increase in PFOA concentration was associated with lower WZ (β = −0.16, 95% confidence interval [CI] = −0.29 to −0.04), LZ (β = −0.16, 95% CI = −0.30 to −0.02), and WLRZ (β = −0.14, 95% CI = −0.28 to −0.00), while PFBA and HFPO-DA were inversely associated with only the WZ (β = −0.11, 95% CI = −0.20 to −0.03 per ln-unit PFBA; β = −0.07, 95% CI = −0.13 to −0.01 per ln-unit HFPO-DA) and WLRZ (β = −0.12, 95% CI = −0.22 to −0.02 per ln-unit PFBA; HFPO-DA: β = −0.07, 95% CI = −0.13 to −0.00 per ln-unit HFPO-DA). In addition, PFNA, PFPeA, and PFHxA were related to WZ (*P* = 0.023, 0.054, and 0.019, respectively) and WLRZ (*P* = 0.009, 0.056, and 0.021, respectively) in a non-linear manner. There was a U-shaped association between maternal sera 6:2 FTS and LZ, while no other PFASs were found to be related to LZ. The estimates (with 95% CI) and significance levels of the linear and non-linear associations between other PFASs and all newborn size parameters are available in Table S3 (Supplementary Materials).

### 3.3 Mixed effect of PFAS exposure and effect modification according to the cord blood FA nutritional status

Overall, the median cord blood FA concentration of the total population was 1116 μg mL^−1^, and those of the upper and lower FA statuses were 1931 and 807 μg mL^−1^, respectively (Supplementary Material Table S4**)**. The demographic characteristics of the mothers of infants with upper versus lower FA statuses were broadly similar (Supplementary Material Table S5). However, the mothers of infants with an upper FA status were significantly older (median age of 31 versus 30, *P* = 0.013) than those of infants with a lower FA status. Moreover, mothers of infants with an upper FA status had lower concentrations of PFOA, 6:2 Cl-PFESA, PFHxA, ADONA, and PFHpA, while they had higher levels of PFOS, HFPO-DA, 6:2 FTS, and PFDoDA compared to those with infants of the lower FA status (Supplementary Material Fig. S2, Table S6).

**Fig. 2:**
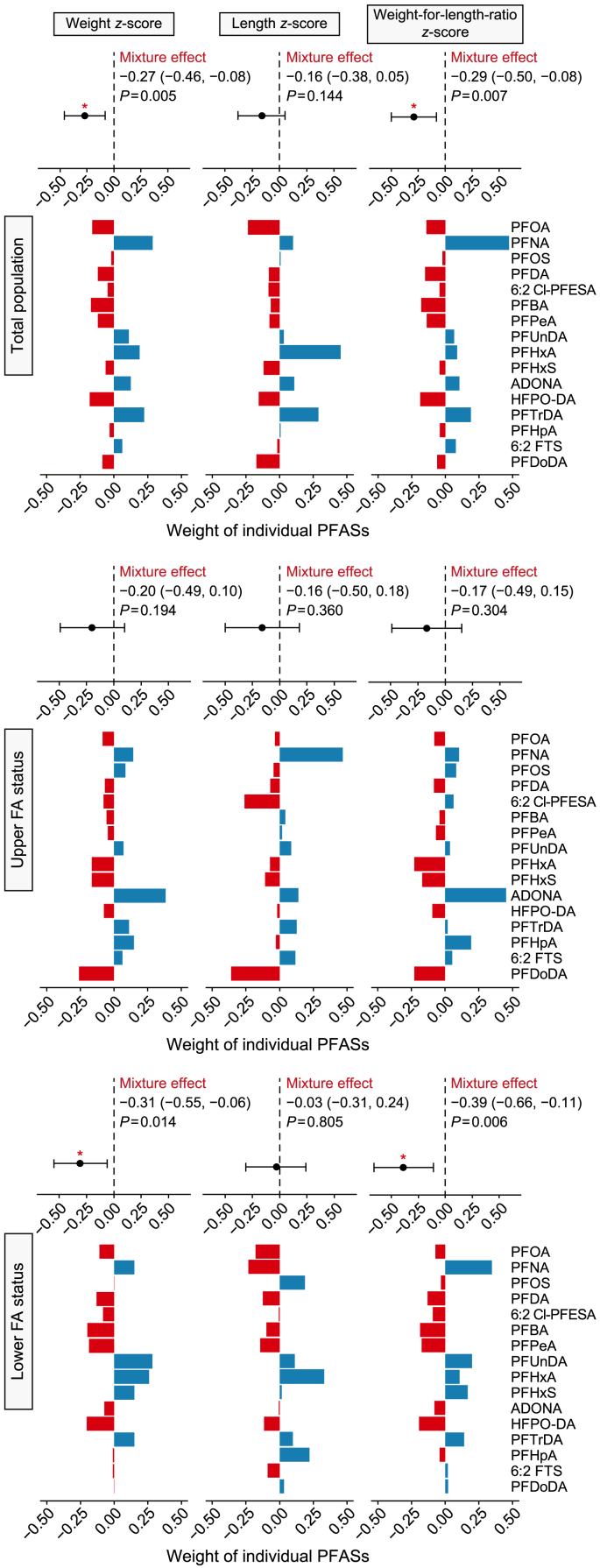
Mixture effect of PFASs with a detection frequency of greater than 60% (*n* = 16) on the newborn size parameters, including weight *z*-score, length *z*-score, and weight-for-length-ratio *z*-score, as determined by quantile-based computation model. Results were adjusted for maternal age, birthweight category (<2500 g; ≥2500, <4000 g; ≥4000 g), maternal pre-pregnancy body mass index, gestational weight gain, sex of newborn, and gestational age at birth. The asterisk (∗) indicates that the mixed effect of PFAS on newborn size parameters is statistically significant (*P* < 0.05).

The mixed effect of PFASs was only observed for WZ and WLRZ at the total population level (WZ: β = −0.27, 95% CI = −0.46 to −0.08; WLRZ: β = −0.29, 95% CI = −0.50 to −0.08) and in infants of the lower FA status (WZ: β = −0.31, 95% CI = −0.55 to −0.06; WLRZ: β = −0.39, 95% CI = −0.66 to −0.11, Fig. 2), while no mixed effect of PFASs was observed for those of the upper FA status. The top contributors to the negative effect were PFOA, PFBA, PFPeA, and HFPO-DA. The BKMR modeling indicated a trend of reduced newborn size with increases in the PFAS concentration, although the results did not reach statistical significance (Supplementary Material Fig. S3). In line with the results derived from QGC modeling, the relative significance levels of PFOA, HFPO-DA, and PFBA were the highest among the 16 tested PFASs for their negative impact on the newborn weight *z*-score, with posterior inclusion probabilities of 0.710, 0.510, and 0.503, respectively (Supplementary Material Table S7).

### 3.4 Cord blood SFAs may attenuate the effect of PFAS exposure

**Fig. 3:**
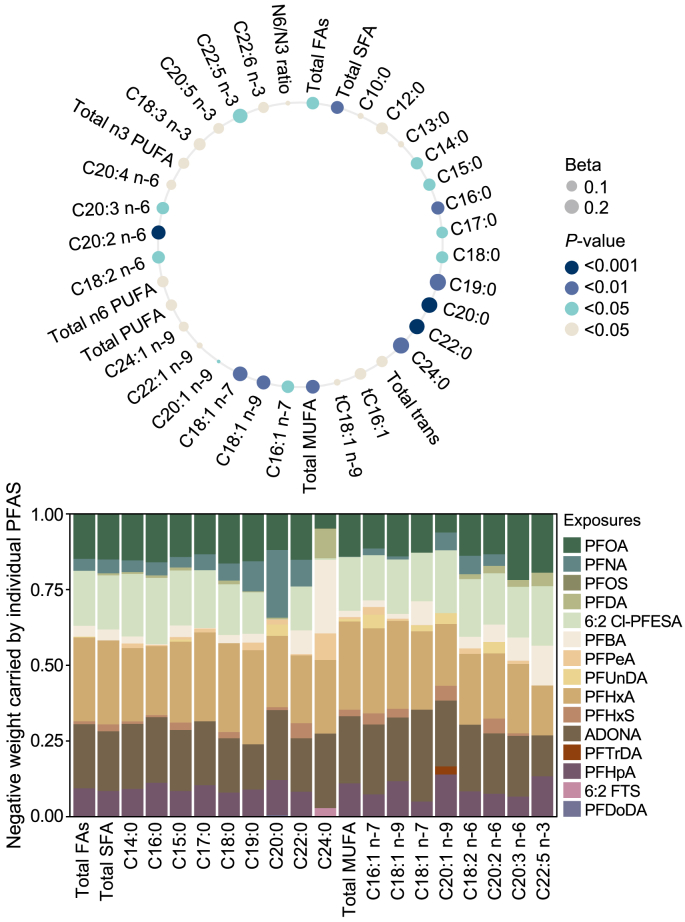
Mixture effect of PFASs with a detection frequency of greater than 60% (*n* = 16) on cord blood fatty acids, as determined by quantile-based g-computation model. Results were adjusted for maternal age, birthweight category (<2500 g; ≥2500, <4000 g; ≥4000 g), maternal pre-pregnancy body mass index, gestational weight gain, sex of newborn, and gestational age at birth. The circle size represents the absolute value of β, the color represents the significance level, and the negative weight carried by individual PFASs is shown in the stack plot.

Inverse associations between mixed PFAS exposure and cord blood FAs were also found for the total FAs (β = −0.14, 95% CI = −0.25 to −0.03), most of the SFAs, and selected MUFAs and polyunsaturated fatty acids (PUFAs) (Fig. 3**,** Supplementary Material Table S8). Cord blood FAs were mostly positively associated with newborn size parameters (Supplementary Material Table S9).

**Fig. 4:**
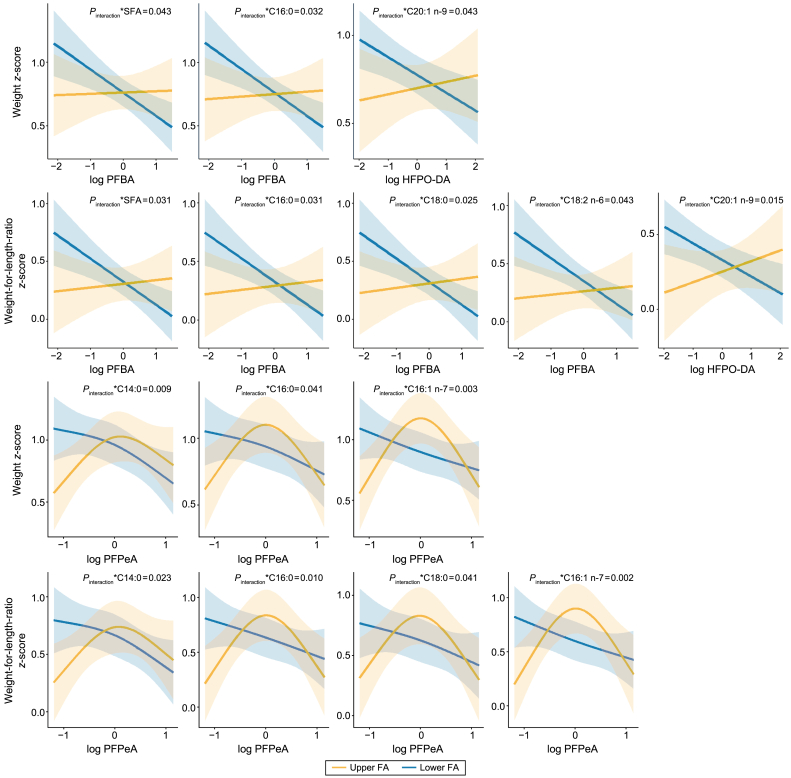
Associations between individual PFAS exposures and newborn size parameters (weight *z*-score, length *z*-score, and weight-for-length-ratio *z*-score) moderated by cord blood fatty acids, determined by generalized linear regression or restrict cubic spline regression for linear (top half) and non-linear (bottom half) associations, respectively. All models were adjusted for maternal age, birthweight category (<2500 g; ≥2500, <4000 g; ≥4000 g), maternal pre-pregnancy body mass index, gestational weight gain, sex of newborn, and gestational age at birth.

Cord blood total SFAs modified the inverse association between maternal PFBA exposure and neonatal WZ (lower SFA: β = −0.19, 95% CI = −0.30 to −0.08; upper SFA: β = −0.01, 95% CI = −0.16 to 0.13; *P*_interaction_ = 0.043) or WLRZ (lower SFA: β = −0.21, 95% CI = −0.33 to −0.08; upper SFA: β = −0.00, 95% CI = −0.15 to 0.15; *P*_interaction_ = 0.031, Fig. 4, Supplementary Material Table S10). For the relationship between HFPO-DA exposure and newborn WZ and WLRZ, MUFA C20:1 n-9 was identified as an effect modifier, with *P*_interaction_ results of 0.043 and 0.015, respectively. In those with a higher SFA status, specifically C14:0, C16:0, and C18:0, there was a bell-shaped relationship between PFPeA and newborn size parameters (WZ: *P*_non-linear_ = 0.030 and 0.004, respectively, for C14:0 and C16:0; WLRZ: *P*_non-linear_ = 0.020, 0.002, and <0.001, Fig. 4**,** Supplementary Material Table S10). The per log-unit increase in gestational exposure to PFPeA was negatively associated with WZ and WLRZ in those of lower cord blood C14:0 (WZ: −0.19 [−0.31 to −0.06], *P* = 0.003; WLRZ: −0.19 [−0.33 to −0.05], *P* = 0.007), C16:0 (WZ: −0.14 [−0.26 to −0.02], *P* = 0.026; WLRZ: −0.16 [−0.29 to −0.25], *P* = 0.023), and C16:1 n-7 (WZ: −0.15 [−0.27 to −0.03], *P* = 0.018; WLRZ: −0.17 [−0.30 to −0.03], *P* = 0.015). No other cord blood FAs moderated the associations between PFAS exposure and newborn size parameters (Supplementary Material Table S11).

## 4 Discussion and conclusion

In this study of 590 mother–infant dyads from the ongoing TIMFEM birth cohort study in central China, we found that gestational exposure to certain PFASs (PFBA, HFPO-DA, and PFPeA) was related to reduced WZ and WLRZ in newborns, especially those of a lower cord blood FA status, but there was null effect in those with a higher FA level. To our knowledge, this is the first report that the fetal nutritional status may ameliorate the adverse effect on newborn size induced by maternal exposure to PFASs.

By utilizing samples from participants of a recently established birth cohort study, we gathered contemporary data on maternal PFAS exposure. In our study, the median levels of PFOA and PFOS were 3.49 and 5.49 ng mL^−1^, respectively, which are far lower than in those maternal at-delivery samples collected from the 1960s (6.19 and 38.2 ng mL^−1^ for PFOA and PFOS, respectively) [33] in the United States. Meanwhile, between 2016 and 2018, in another birth cohort in Zhejiang, China, maternal serum PFOA and PFOS levels of 7.91 and 11.61 ng mL^−1^ were reported in the first trimester [19], double the levels reported in our study. A recent report suggests that maternal PFOA and PFOS generally decline throughout pregnancy at 4–5% per month [34]; however, this cannot explain the magnitude of differences observed here. Instead, the discrepancies observed are likely due to the gradual phase-out of legacy PFASs following inclusion in the Stockholm Convention and differences in the habitual or preferential intake of seafood products [35,36], a dominant source of dietary PFAS exposure, as Zhejiang Province is located on the east coast of China. Although legacy PFASs remain the most abundant PFASs detected in maternal serum, the effect of PFASs on the newborn size mostly relates to three newly emerging carboxylic-group-containing PFASs: PFBA, HFPO-DA, and PFPeA. These short-chain homologs were of a similar concentration, at approximately 0.5 ng mL^−1^, which was only one-tenth of the concentration of PFOS measured but demonstrated negative associations with the newborn size across our single and mixed models.

Gestational exposure to PFASs, especially PFOA, PFBA, HFPO-DA, and PFPeA, was associated with a reduced newborn WZ and WLRZ. Only maternal sera 6:2 FTS was correlated with a reduced birth LZ, as found in the single-analyte model, while a null association was found in the PFAS mixed model, along with null associations for the other PFASs. Similar findings were reported by Zheng et al., where the authors measured 27 PFASs from maternal serum and found gestational exposure to both legacy and emerging PFASs to be strongly related to reduced birth weight (PFOA:β = −0.233; PFOS: β = −0.323; PFHxS: β = −0.292; PFHpS: β = −0.239; PFNA: β = −0.239) with similar effect sizes, but not with the length measured at birth [21]. Moreover, our recent work demonstrated that gestational exposure to a human-relevant dose of PFHxS induces intrauterine growth restriction in a mouse model [37], providing the first evidence for the developmental toxicity of newly emerging PFASs. In contrast, through a nested case–control study within the LIFECODES cohort in Boston, it was found that none of the nine measured PFASs (N-MeFOSAA, PFDA, PFHpA, PFHxS, PFNA, PFOA, PFOS, FOSA, and PFUnDA) were associated with WZ or the odds of SGA [10]. Nonetheless, Ji et al. demonstrated that placental PFASs, but not maternal serum, were associated with a reduced birth weight (PFOA: β = −103.8; PFNA: β = −99.9; PFDA: β = −65.7; 6:2 Cl-PFESA: β = −80.0) [38]. Although inconsistent associations between gestational exposure to PFASs and newborn size parameters have been observed, these may be attributable to research design factors such as using different matrices [39]. In general, the collective evidence pointed toward a trend of reduced birth size with an increase in gestational exposure to PFASs.

Additionally, our study reports that gestational exposure to PFASs is associated with reduced cord blood total FAs and most SFAs and MUFAs. We further demonstrated that the associations between exposure to PFBA, HFPO-DA, and PFPeA and a reduced birth size were more pronounced in those of a lower FA status. The potential influence of PFAS exposure on fetal growth was moderated by cord blood FAs, especially SFAs and MUFAs, which may be due to their primary biological functions as energy substrates. Different types of FAs share a similar fate in the human body of being catabolized for energy production through multiple rounds of β-oxidation or being incorporated into the phospholipid bilayer of cell membranes. However, long-chain PUFAs may be further metabolized into bioactive molecules through enzymatic and/or non-enzymatic pathways [40], which channels signaling pathways between cells [41], regulates inflammatory responses [42], and induces cellular senescence [43]. A series of enzymes and FA transporters located on the placenta assist in the hydrolysis and transfer of complex lipids and non-esterified FAs from the maternal circulation across the placenta for normal growth and development of the fetus [44]. While the fundamental role of SFAs and MUFAs is as energy substrates, long-chain PUFAs, especially docosahexaenoic acid (DHA, C22:6 n-3), are reserved for the development of the brain and retinas [45].

Dysfunction of the PPAR signaling pathway of the placenta induced by PFAS exposure has been proposed to be the underlying cause of reduced newborn size [46]. Using untargeted metabolomic analysis, a recent study identified correlations between the early gestational PFAS concentration and several pathways regulating FA metabolism, especially FA oxidation, in the umbilical cord [47]. However, the authors acknowledged that these exploratory analyses lacked important health-related endpoints and suggested that birthweight should be a priority for further investigation [47]. In another study from 2015, Kishi and colleagues reported that gestational PFOS exposure was related to disturbed maternal FA metabolism. This was indicated by lowered levels of C16:0, C16:1 n-7, C18:1 n-9, and essential FAs, as well as a lower birth weight in female infants [48]. Our study and a recent report of an adverse effect of gestational exposure to legacy PFASs on birth outcomes, which was attenuated by the maternal folate status [33], suggests that nutritional intervention may be feasible in mitigating the negative impact of environmental pollutants on human health.

### 4.1 Strengths and limitations

This study provides comprehensive and contemporary data regarding PFAS exposure burdens in pregnant mothers, further to the regulatory restriction of many legacy and emerging PFASs. Past reports have not extensively explored many of the short-chain emerging PFASs. However, we revealed possible detrimental effects on birth outcomes, which is critical in informing public health. By performing both single-analyte and mixed-model analyses, we revealed that certain PFASs consistently demonstrated inverse associations with newborn size parameters. This information should guide relevant governing bodies in determining further regulatory control measures for these PFASs. Lastly, by incorporating the analysis of cord blood FAs, we showed that nutritional interventions might be useful in mitigating the adverse effects of PFASs on human health, which warrants further research and possibly interventional trials for confirmation. However, it is important to note the limitations of this study. Although this study was based on an ongoing prospective birth cohort, maternal serum samples were collected before delivery. Furthermore, the cross-sectional nature of this study limited our interpretation regarding the longitudinal changes throughout pregnancy. It did not allow for early prediction of adverse birth outcomes based on maternal exposure. Additionally, it has been shown that the concentration of legacy PFASs declines with the progress of pregnancy [34], which was something we did not investigate. So, the variability of emerging PFASs throughout gestation requires further investigation. Furthermore, different matrices were used for PFASs' measurement and FAs’ profiling. Using the same sample matrix might improve data consistency; however, we intended to understand the effect modification of the fetal nutritional status concerning gestational exposure to environmental factors, for which different matrices were appropriate.

### 4.2 Implications

With the gradual phase-out of conventional PFASs and their replacement with short-chain emerging congeners, the related health consequences have received increasing attention. Our study first analyzed gestational exposure to 30 conventional and newly emerging PFASs and their relations to a reduced newborn size. We further investigated the possibility that nutritional factors impacting the fetal FA status could moderate or mitigate the adverse effects of environmental exposure on human health. Altogether, our work revealed the potential developmental toxicity of carboxylic-group-containing PFASs, which was not well characterized before. Therefore, our work has several important implications for both research and policy translation.

First, newly emerging PFASs, especially those containing a carboxylic group, even at a low dosage might pose greater harm to fetal health than the legacy PFASs. Our work revealed that legacy PFASs remain the most abundant PFASs measured in maternal serum, while PFBA, HFPO-DA, and PFPeA were only present at one-sixth to one-tenth of their concentrations. However, in the single-analyte and mixed-model analyses, these emerging PFASs demonstrated adverse effects on fetal outcomes similar to the conventional congeners. Whether these observed effects were due to an enhanced placental transfer efficiency or the metabolic fate of these substances within the human body remains to be explored. Nevertheless, these results call for immediate attention to these emerging PFASs and rigorously studied their developmental toxicities in experimental animals and population settings.

Second, genetic, nutritional, and environmental exposure represent the three most important factors in human health and diseases. In our daily lives, exposure to environmental toxicants and nutrition are often intertwined, as foods are not only a source of nutritional elements but may also contain trace amounts of toxicants due to their packaging, processing, and use of water sources. Previous research has largely focused on the isolated effect of environmental exposure or nutrition on human health outcomes. It has neglected that these two might interact within the human body and/or contribute to the progress of disease simultaneously [49], which highlights why they should be studied concurrently.

Last, many forms of environmental exposure have metabolism-disrupting potential [50], and PFASs can interfere with lipid metabolism through PPAR signaling pathways. To our knowledge, this study is the first to report a higher fetal FA status, especially in SFAs and MUFAs, moderating the effect of PFASs on newborn size parameters. Although the underlying mechanism remains to be investigated, SFAs and MUFAs are primary energy substrates for the developing fetus, while PUFAs assume other important roles, including the development of the brain and retinas. Fetal FAs are mainly sourced from maternal circulation, so the potential for an improved maternal nutritional status during pregnancy to ameliorate the adverse effects of environmental pollutants and thereby improve maternal and fetal outcomes should be explored in future studies. In that direction, our work provides important evidence that a favorable nutritional status may counteract the adverse effects of environmental insults, offering an important mitigation strategy for disease prevention and control.

In conclusion, in this cohort of 590 mother–infant pairs from China, we found that gestational PFAS exposure was negatively associated with the newborn size, with PFOA, PFBA, HFPO-DA, and PFPeA being the main effect drivers. By dividing the population based on the cord blood FA status, we found that the above associations were only sound in those of a lower FA status. Furthermore, we found that the effect of PFAS exposure on the newborn size was moderated by cord blood SFAs and MUFAs. Based on our findings, we proposed that nutritional approaches may be feasible in ameliorating the adverse health effects of environmental PFAS exposure.

## CRediT authorship contribution statement

**Chang Gao:** Writing - Review & Editing, Writing - Original Draft, Visualization, Methodology, Formal Analysis, Conceptualization. **Lin Luo:** Writing - Review & Editing, Methodology, Formal Analysis. **Yijun Fan:** Writing - Review & Editing, Resources, Project Administration, Data Curation. **Liyan Guo:** Writing - Review & Editing, Methodology, Data Curation. **Lijuan Guo:** Writing - Review & Editing, Methodology, Data Curation. **Lin Tao:** Writing - Review & Editing, Resources, Methodology. **Fangbiao Tao:** Writing - Review & Editing, Resources, Methodology. **De-Xiang Xu:** Writing - Review & Editing, Resources, Project Administration. **Robert A. Gibson:** Writing - Review & Editing, Writing - Original Draft, Methodology. **Maria Makrides:** Writing - Review & Editing, Methodology. **Hua Wang:** Writing - Review & Editing, Supervision, Resources, Project administration, Conceptualization. **Yichao Huang:** Writing - Review & Editing, Writing - Original Draft, Supervision, Resources, Project Administration, Methodology, Funding Acquisition, Data Curation, Conceptualization.

## Declaration of competing interest

The authors declare that they have no known competing financial interests or personal relationships that could have appeared to influence the work reported in this paper.
