# Supplementary material for: Gestational PFAS exposure and newborn size: The modifying effect of cord blood fatty acids
Source: Environ Sci Ecotechnol. 2024 Aug 3;22:100476. doi: 10.1016/j.ese.2024.100476 (PMC11369372; doi:10.1016/j.ese.2024.100476)
Supplement: Multimedia component 1 [file mmc1.docx]

**SUPPORTING INFORMATION**

**Gestational PFAS Exposure and Newborn Size: The Modifying Effect of Cord Blood Fatty Acids**

Chang Gao ^a, b, c, d, 1^, Lin Luo ^a, b, c, d, 1^, Yijun Fan ^e,^ ^1^, Liyan Guo ^a, b, c, d^, Lijuan Guo ^a, b, c, d^, Lin Tao ^a, b, c, d^, Fangbiao Tao ^d,^ ^f^, De-Xiang Xu ^a, b, c, d^, Robert A Gibson ^g^, Maria Makrides ^g^, Hua Wang ^a, b, c, d, *^, Yichao Huang ^a, b, c, d, e, h, *^

**Affiliations:**

*^a^* *Department of Toxicology, School of Public Health, Anhui Medical University, Hefei, 230031, China*

*^b^ Key Laboratory of Environmental Toxicology of Anhui Higher Education Institutes, Anhui Medical University, Hefei, 230031, China*

*^c^ Center for Big Data and Population Health of IHM, Anhui Medical University, Hefei, 230031, China*

*^d^ MOE Key Laboratory of Population Health Across Life Cycle, Anhui Medical University, Hefei, 230031, China*

*^e^ Department of Gynecology and Obstetrics, the Second Affiliated Hospital of Anhui Medical University, Hefei, 230601, China*

*^f^ Department of Maternal & Child and Adolescent Health, School of Public Health, Anhui Medical University, Hefei, 230031, China*

*^g^ SAHMRI Women and Kids, South Australian Health and Medical Research Institute, North Adelaide, 5000, South Australia, Australia*

*^h^ Clinical Research Center, Suzhou Hospital of Anhui Medical University, Suzhou,* *234099, China*

*** Corresponding authors:**

Dr **Yichao Huang**, Department of Toxicology, School of Public Health, Anhui Medical University, 81 Meishan Rd, Hefei 230032, China. E-mail: [yichao.huang@ahmu.edu.cn](mailto:yichao.huang@ahmu.edu.cn)

Dr. **Hua Wang**, Department of Toxicology, School of Public Health, Anhui Medical University, 81 Meishan Rd, Hefei 230032, China. E-mail: [wanghuadev@126.com](mailto:wanghuadev@126.com)

*^¶^* Indicates equal contribution to this work.

| **Table of Content** | | |
| --- | --- | --- |
| **Text/Table/Figure number** | **Description/title/legends** | **Page number** |
| **Table S1** | List of 30 PFASs measured in present study with names and CAS number. | S4 |
| **Table S2** | Distribution of 30 PFASs measured in maternal serum in the present study (ng/mL). | S5 |
| **Table S3** | Significant level of the association between individual PFASs exposure and newborn size parameters, determined by generalized linear regression or restricted cubic spline regression. All models were adjusted for maternal age, birthweight category (<2500g, ≥2500g & <4000g, ≥4000g), maternal pre-pregnancy body mass index, gestational weight gain, sex of newborn, gestational age at birth. | S6 |
| **Table S4** | Distribution of cord blood fatty acid composition (μg/mL) in total population and those of upper and lower fatty acid status. | S7 |
| **Table S5** | Demographic characteristics of mother-infant dyads with different cord blood fatty acid concentrations. | S8 |
| **Table S6** | Comparison of maternal serum PFASs level (ng/mL) in neonates of higher versus lower cord blood total fatty acids status. | S10 |
| **Table S7** | Posterior Inclusion Probability of sixteen PFASs included in the Bayesian Kernal Machine Regression modelling for analysing the PFASs mixture effect on newborn size parameters, adjusted for maternal age, maternal birthweight category (<2500g, ≥2500g & <4000g, ≥4000g), maternal pre-pregnancy body mass index, gestational weight gain, sex of newborn, gestational age at birth. | S11 |
| **Table S8** | Mixture of all PFASs with detection frequency of greater than 60% (n=16) on cord blood fatty acid level as determined by quantile-based g-computation model, weight of individual exposures only shown for those significantly affected fatty acids. | S12 |
| **Table S9** | Association between selected cord blood fatty acids (log transformed) and newborn size parameters determined by multivariate linear model, adjusted for maternal age, maternal birthweight category (<2500g, ≥2500g & <4000g, ≥4000g), maternal pre-pregnancy body mass index, gestational weight gain, sex of newborn, gestational age at birth. | S14 |
| **Table S10** | Association between selected PFASs and newborn size parameters were modified by selected fatty acids (upper versus lower), coefficient and 95% confidence interval along with *P* values were reported for multiple linear models, while only *P* values were reported for restricted cubic spline model. All models were adjusted for maternal age, maternal birthweight category (<2500g, ≥2500g & <4000g, ≥4000g), maternal pre-pregnancy body mass index, gestational weight gain, sex of newborn, gestational age at birth. | S15 |
| **Table S11** | Significant level of the effect modification by cord blood fatty acids on the association between individual PFASs exposure and newborn size parameters, determined by generalized linear regression or restricted cubic spline regression. All models were adjusted for maternal age, maternal birthweight category (<2500g, ≥2500g & <4000g, ≥4000g), maternal pre-pregnancy body mass index, gestational weight gain, sex of newborn, gestational age at birth. | S16 |
| **Figure S1** | Spearman correlations between all 16 PFASs with detection frequency above 60%. | S17 |
| **Figure S2** | Comparison of maternal serum PFASs level (ng/mL) in neonates of higher versus lower cord blood total fatty acids level (μg/mL). Starts indicating significance level, where **P*<0.05, ***P*<0.01, ****P*<0.001. | S18 |
| **Figure S3** | Figure S3. Mixture effect of PFASs (detection frequency ≥60%, n=16) on newborn size parameters (Weight Z-Score, Length Z-Score and Weight-for-Length Ratio Z-score) in the total population and the population stratified by cord blood fatty acid status, by Bayesian Kernal Regression modelling. The model was adjusted for maternal age, maternal birthweight category (<2500g, ≥2500g & <4000g, ≥4000g), maternal pre-pregnancy body mass index, gestational weight gain, sex of newborn, gestational age at birth. | S19 |

**Table S1.** List of 30 PFASs measured in present study with names and CAS number.

| Full Name | Abbreviated Names | CAS number |
| --- | --- | --- |
| Perfluorobutanoic acid | PFBA | 375-22-4 |
| Perfluoropentanoic acid | PFPeA | 2706-90-3 |
| Perfluorohexanoic acid | PFHxA | 307-24-4 |
| Perfluoroheptanoic acid | PFHpA | 375-85-9 |
| Perfluorooctanoic acid | PFOA | 335-67-1 |
| Perfluorononanoic acid | PFNA | 375-95-1 |
| Perfluorodecanoic acid | PFDA | 335-76-2 |
| Perfluoroundecanoic acid | PFUnDA | 2058-94-8 |
| Perfluorododecanoic acid | PFDoDA | 307-55-1 |
| Perfluorotridecanoic acid | PFTrDA | 72629-94-8 |
| Perfluorotetradecanoic acid | PFTeDA | 376-06-7 |
| Perfluorohexadecanoic acid | PFHxDA | 67905-19-5 |
| Perfluorooctadecanoic acid | PFODA | 16517-11-6 |
| dodecafluoro-3H-4,8dioxanonanoate | ADONA |  |
| hexafluoropropylene oxide dimer acid | HFPO-DA | 13252-13-6 |
| 8:2 fluorotelomer acid | 8:2 FTA | 70887-84-2 |
| Perfluorobutanesulfonic acids | PFBS | 29420-49-3 |
| Perfluorohexanesulfonic acid | PFHxS | 82382-12-5 |
| Perfluoroheptanesulfonic acid | PFHpS |  |
| Perfluorooctane sulfonate | PFOS | 4021-47-0 |
| 6:2 chlorinated polyfluorinated ether sulfonate | 6:2 Cl-PFESA | 73606-19-6 |
| Perfluorodecanesulfonic acid | PFDS | 335-77-3 |
| 6:2 fluorotelomer sulfonate | 6:2 FTS | 29420-49-3 |
| 8:2 fluorotelomer sulfonate | 8:2 FTS | 39108-34-4 |
| Perfluorooctane sulfonamide | FOSA | 754-91-6 |
| n-methyl-perfluorooctane sulfonamide | N-MeFOSA | 31506-32-8 |
| n-ethylperfluorooctane sulfonamide | N-EtFOSA | 4151-50-2 |
| n-methylperfluorooctane sulfonamidoacetates | N-MeFOSAA | 2991-50-6 |
| n-ethylperfluorooctane sulfonamidoacetates | N-EtFOSAA | 2355-31-9 |
| 8:2 polyfluoroalkyl phosphate diester | 8:2 diPAP | 678-41-1 |

CAS: Chemical Abstracts Service

**Table S2.** Distribution of 30 PFASs measured in maternal serum in the present study (ng/mL).

| **PFASs** | **Recovery^a^** | **Recovery^b^** | **DF** | **LOQ** | **Min** | **25^th^** | **Median** | **Mean** | **75^th^** | **Max.** |
| --- | --- | --- | --- | --- | --- | --- | --- | --- | --- | --- |
| Total PFASs | - | - | 100.0 | **-** | 6.91 | 14.9 | 19.2 | 20.8 | 25.4 | 57.2 |
| PFOA | 84.4±2.6% | 111.6±9.5% | 100.0 | 0.084 | 0.726 | 2.84 | 3.97 | 4.72 | 5.47 | 17.4 |
| PFOS | 84.3±0.9% | 101.3±1.0% | 100.0 | 0.062 | 0.396 | 3.25 | 5.49 | 6.36 | 7.97 | 28.2 |
| PFNA | 97.7±1.9% | 63.9±2.3% | 100.0 | 0.092 | 0.098 | 0.588 | 0.987 | 1.21 | 1.56 | 5.49 |
| PFDA | 98.9±2.3% | 57.4±6.9% | 100.0 | 0.081 | 0.106 | 0.570 | 0.950 | 1.22 | 1.64 | 7.15 |
| 6:2 Cl-PFESA | 103.4±0.5% | 40.9±3.5% | 100.0 | 0.015 | 0.118 | 0.682 | 1.14 | 1.46 | 1.91 | 7.56 |
| PFBA | 106.0±5.8% | 74.4±2.2% | 99.8 | 0.095 | <LOQ | 0.311 | 0.540 | 0.725 | 1.06 | 2.50 |
| PFPeA | 88.1±2.1% | 120.8±6.4% | 99.8 | 0.098 | <LOQ | 0.352 | 0.593 | 0.735 | 1.07 | 2.03 |
| PFUnDA | 79.0±5.0% | 56.3±26.6% | 99.8 | 0.094 | <LOQ | 0.427 | 0.822 | 1.02 | 1.36 | 6.24 |
| PFHxA | 86.4±0.8% | 91.5±2.3% | 95.6 | 0.050 | <LOQ | 0.142 | 0.257 | 0.408 | 0.527 | 3.05 |
| PFHxS | 92.1±7.9% | 77.1±3.3% | 93.2 | 0.071 | <LOQ | 0.614 | 1.04 | 1.18 | 1.58 | 5.84 |
| ADONA | 95.1±0.6% | 88.5±2.1% | 91.2 | 0.002 | <LOQ | 0.005 | 0.011 | 0.019 | 0.022 | 0.241 |
| HFPO-DA | 99.7±4.9% | 107.3±5.5% | 84.6 | 0.073 | <LOQ | 0.214 | 0.421 | 0.612 | 0.911 | 3.03 |
| PFTrDA | 117.5±17.8% | 65.2±2.7% | 84.4 | 0.042 | <LOQ | 0.061 | 0.113 | 0.146 | 0.193 | 0.944 |
| PFHpA | 99.8±3.0% | 88.2±1.3% | 82.4 | 0.023 | <LOQ | 0.041 | 0.098 | 0.159 | 0.187 | 2.56 |
| 6:2FTS | 139.6±4.8% | 98.2±3.2% | 73.4 | 0.035 | <LOQ | <LOQ | 0.091 | 0.125 | 0.175 | 0.869 |
| PFDoDA | 45.6±6.2% | 52.0±4.3% | 67.5 | 0.062 | <LOQ | <LOQ | 0.095 | 0.142 | 0.180 | 1.41 |
| 8:2FTS | 103.3±5.0% | 58.3±1.7% | 48.1 | 0.020 | <LOQ | <LOQ | <LOQ | 0.046 | 0.052 | 0.996 |
| PFDS | 24.9±1.6% | 29.3±3.4% | 47.5 | 0.014 | <LOQ | <LOQ | <LOQ | 0.034 | 0.035 | 0.466 |
| PFBS | 92.3±3.6% | 91.5±5.6% | 47.1 | 0.039 | <LOQ | <LOQ | <LOQ | 0.054 | 0.068 | 0.391 |
| PFHpS | 101.1±2.7% | 84.8±2.8% | 43.4 | 0.048 | <LOQ | <LOQ | <LOQ | 0.063 | 0.077 | 0.456 |
| PFODA | 45.6±6.2% | 52.0±4.3% | 41.4 | 0.002 | <LOQ | <LOQ | <LOQ | 0.030 | <LOQ | 0.688 |
| N-MeFOSA | 90.3±2.8% | 110.3±16.7% | 36.4 | 0.008 | <LOQ | <LOQ | <LOQ | 0.032 | 0.033 | 0.588 |
| 8:2 FTA | 95.8±1.1% | 66.5±2.7% | 31.0 | 0.019 | <LOQ | <LOQ | <LOQ | 0.026 | 0.024 | 0.686 |
| N-EtFOSA | 50.3±4.2% | 55.8±3.3% | 30.3 | 0.017 | <LOQ | <LOQ | <LOQ | 0.037 | 0.027 | 1.17 |
| PFHxDA | 68.2±4.9% | 33.2±4.4% | 29.5 | 0.013 | <LOQ | <LOQ | <LOQ | 0.042 | 0.031 | 1.02 |
| N-MeFOSAA | 97.8±7.1% | 115.5±9.5% | 28.3 | 0.015 | <LOQ | <LOQ | <LOQ | 0.028 | 0.021 | 1.20 |
| PFTeDA | 45.5±1.5% | 46.8±2.6% | 25.1 | 0.061 | <LOQ | <LOQ | <LOQ | 0.075 | 0.057 | 2.78 |
| N-EtFOSAA | 76.9±5.9% | 43.3±9.0% | 21.5 | 0.027 | <LOQ | <LOQ | <LOQ | 0.030 | <LOQ | 0.290 |
| 8:2diPAP | 34.3±7.0% | 30.4±5.2% | 14.1 | 0.028 | <LOQ | <LOQ | <LOQ | 0.029 | <LOQ | 0.358 |
| FOSA | 60.5±5.1% | 62.3±4.6% | 8.8 | 0.012 | <LOQ | <LOQ | <LOQ | <LOQ | <LOQ | 0.114 |

DF: detection frequency; LOQ: limit of quantification. Superscript a refers to blank spiking and b indicates matrix spiking tests.

**Table S3.** Significant level of the association between individual PFASs exposure and newborn size parameters, determined by generalized linear regression or restricted cubic spline regression. All models were adjusted for maternal age, birthweight category (<2500g, ≥2500g & <4000g, ≥4000g), maternal pre-pregnancy body mass index, gestational weight gain, sex of newborn, gestational age at birth.

|  | **Weight Z-Score** | | | | **Length Z-Score** | | | | **Weight-for-Length-Ratio Z-Score** | | | |
| --- | --- | --- | --- | --- | --- | --- | --- | --- | --- | --- | --- | --- |
|  | β | 95% CI | *P*_linear_ | *P*_non-linear_ | β | 95% CI | *P*_linear_ | *P*_non-linear_ | β | 95% CI | *P*_linear_ | *P*_non-linear_ |
| PFOA | **-0.16** | **(-0.29, -0.04)** | **0.011** | **0.018** | **-0.16** | **(-0.30, -0.02)** | **0.029** | 0.066 | **-0.14** | **(-0.28, -0.00)** | **0.048** | **0.032** |
| PFNA | -0.06 | (-0.15, 0.03) | 0.220 | **0.023** | -0.05 | (-0.16, 0.05) | 0.295 | 0.801 | -0.04 | (-0.14, 0.06) | 0.465 | **0.009** |
| PFOS | -0.07 | (-0.17, 0.03) | 0.160 | 0.395 | -0.08 | (-0.19, 0.03) | 0.150 | 0.554 | -0.05 | (-0.16, 0.05) | 0.314 | 0.267 |
| PFDA | -0.07 | (-0.16, 0.02) | 0.108 | 0.446 | -0.06 | (-0.16, 0.03) | 0.202 | 0.342 | -0.07 | (-0.17, 0.03) | 0.149 | 0.236 |
| 6:2 Cl-PFESA | -0.08 | (-0.17, 0.01) | 0.065 | 0.237 | -0.07 | (-0.17, 0.03) | 0.194 | 0.722 | -0.08 | (-0.18, 0.02) | 0.102 | 0.210 |
| PFBA | **-0.11** | **(-0.20, -0.03)** | **0.010** | 0.770 | -0.03 | (-0.13, 0.07) | 0.531 | 0.536 | **-0.12** | **(-0.22, -0.02)** | **0.015** | 0.927 |
| PFPeA | -0.08 | (-0.18, 0.01) | 0.088 | **0.054** | -0.05 | (-0.16, 0.06) | 0.377 | 0.804 | -0.09 | (-0.20, 0.01) | 0.092 | **0.056** |
| PFUnDA | -0.02 | (-0.10, 0.06) | 0.565 | 0.918 | -0.03 | (-0.12, 0.06) | 0.522 | 0.566 | -0.03 | (-0.12, 0.06) | 0.528 | 0.887 |
| PFHxA | 0.02 | (-0.04, 0.09) | 0.487 | **0.019** | 0.06 | (-0.02, 0.13) | 0.137 | 0.118 | 0.01 | (-0.07, 0.08) | 0.877 | **0.021** |
| PFHxS | -0.03 | (-0.09, 0.04) | 0.423 | 0.759 | -0.04 | (-0.11, 0.04) | 0.331 | 0.276 | -0.02 | (-0.09, 0.05) | 0.587 | 0.437 |
| ADONA | -0.00 | (-0.06, 0.05) | 0.883 | 0.119 | 0.01 | (-0.05, 0.08) | 0.720 | 0.309 | -0.01 | (-0.07, 0.06) | 0.816 | 0.156 |
| HFPO-DA | **-0.07** | **(-0.13, -0.01)** | **0.019** | 0.591 | -0.05 | (-0.12 0.02) | 0.145 | 0.762 | **-0.07** | **(-0.13, -0.00)** | **0.041** | 0.622 |
| PFTrDA | -0.01 | (-0.09, 0.07) | 0.766 | 0.322 | -0.01 | (-0.10, 0.08) | 0.835 | 0.523 | -0.01 | (-0.10, 0.08) | 0.809 | 0.306 |
| PFHpA | -0.04 | (-0.10, 0.01) | 0.141 | 0.339 | -0.01 | (-0.08, 0.05) | 0.745 | 0.251 | -0.05 | (-0.11, 0.02) | 0.165 | 0.468 |
| 6: 2FTS | 0.01 | (-0.06, 0.08) | 0.827 | 0.094 | 0.00 | (-0.08, 0.08) | 0.914 | **0.030** | 0.01 | (-0.07, 0.09) | 0.855 | 0.407 |
| PFDoDA | -0.07 | (-0.15, 0.02) | 0.124 | 0.831 | -0.09 | (-0.18, 0.01) | 0.068 | 0.576 | -0.05 | (-0.14, 0.04) | 0.289 | 0.638 |

**Table S4.** Distribution of cord blood fatty acid composition (μg/mL) in total population and those of upper and lower fatty acid status.

|  | **Whole population** | | **Upper FA status** | | **Lower FA status** | |
| --- | --- | --- | --- | --- | --- | --- |
|  | **(n=590)** | | **(n=197)** | | **(n=393)** | |
| Total FA | 1116 | (722, 1741) | 1931 | (1741, 2245) | 807 | (658, 1116) |
| Total SFA | 428.17 | (284.86, 686.65) | 765.20 | (686.4,901.7) | 315.06 | (256.80, 428.17) |
| C10:0 | 0.14 | (0.09, 0.20) | 0.20 | (0.16, 0.28) | 0.11 | (0.07, 0.15) |
| C12:0 | 0.50 | (0.36, 0.71) | 0.72 | (0.58,0.97) | 0.42 | (0.31, 0.56) |
| C13:0 | 0.03 | (0.02, 0.04) | 0.04 | (0.03,0.05) | 0.02 | (0.02, 0.03) |
| C14:0 | 5.38 | (3.52, 8.59) | 10.11 | (8.29,12.41) | 4.09 | (3.04, 5.53) |
| C15:0 | 1.09 | (0.75, 1.66) | 1.89 | (1.55,2.27) | 0.85 | (0.67, 1.12) |
| C16:0 | 291.39 | (190.03, 460.68) | 534.70 | (454.1,627.1) | 214.88 | (170.87, 291.39) |
| C17:0 | 1.79 | (1.30, 2.69) | 2.96 | (2.56,3.60) | 1.42 | (1.14, 1.81) |
| C18:0 | 118.86 | (79.45, 188.10) | 209.50 | (188.0,242.9) | 87.02 | (72.80, 118.86) |
| C19:0 | 0.59 | (0.17, 0.85) | 0.90 | (0.80,1.08) | 0.20 | (0.14, 0.59) |
| C20:0 | 2.15 | (1.42, 3.18) | 3.30 | (2.67,4.03) | 1.60 | (1.25, 2.20) |
| C22:0 | 3.32 | (2.15, 4.94) | 5.45 | (4.20,7.03) | 2.44 | (1.88, 3.43) |
| C24:0 | 3.00 | (2.11, 4.52) | 4.69 | (3.59,6.32) | 2.43 | (1.87, 3.31) |
| Total Trans | 3.76 | (2.57, 5.75) | 5.97 | (1.20,8.03) | 3.52 | (2.81, 4.83) |
| tC16:1 | 3.29 | (2.18, 5.27) | 5.35 | (0.50,7.35) | 3.09 | (2.38, 4.13) |
| tC18:1 n-9 | 0.49 | (0.29, 0.70) | 0.62 | (0.49,0.87) | 0.40 | (0.25, 0.58) |
| Total MUFA | 271.74 | (173.07, 431.32) | 497.80 | (423.1,615.2) | 194.59 | (156.30, 271.74) |
| C16:1 n-7 | 25.19 | (16.74, 37.01) | 43.81 | (33.95,52.89) | 18.91 | (14.43, 25.46) |
| C18:1 n-9 | 209.52 | (129.77, 338.16) | 385.40 | (332.1,490.5) | 146.53 | (118.04, 209.52) |
| C18:1 n-7 | 27.96 | (17.80, 43.98) | 51.27 | (39.70,62.47) | 20.22 | (15.84, 29.20) |
| C20:1 n-9 | 0.85 | (0.61, 1.21) | 1.29 | (1.09,1.54) | 0.68 | (0.54, 0.90) |
| C22:1 n-9 | 1.78 | (1.11, 2.16) | 1.47 | (1.03,1.95) | 1.92 | (1.36, 2.20) |
| C24:1 n-9 | 6.06 | (4.06, 8.42) | 7.23 | (5.35,11.21) | 5.33 | (3.62, 7.56) |
| Total PUFA | 400.02 | (257.26, 591.27) | 662.10 | (580.4,763.5) | 289.01 | (227.19, 400.02) |
| Total n6 PUFA | 352.49 | (229.45, 519.71) | 589.50 | (519.0,683.8) | 255.87 | (198.65, 352.49) |
| C18:2 n-6 | 130.50 | (86.12, 198.29) | 226.60 | (192.0,277.5) | 97.69 | (75.09, 130.50) |
| C20:2 n-6 | 5.26 | (3.59, 7.49) | 8.36 | (6.87,10.29) | 4.12 | (3.17, 5.39) |
| C20:3 n-6 | 33.08 | (20.42, 51.88) | 57.61 | (47.64,70.05) | 23.37 | (18.00, 33.08) |
| C20:4 n-6 | 178.95 | (113.49, 264.32) | 289.40 | (250.0,339.0) | 129.98 | (99.83, 180.50) |
| Total n3 PUFA | 41.14 | (28.75, 59.38) | 69.18 | (55.61, 84.79) | 32.62 | (24.98, 41.98) |
| C18:3 n-3 | 1.45 | (0.97, 2.23) | 2.39 | (1.70, 3.42) | 1.14 | (0.81, 1.68) |
| C20:5 n-3 | 1.54 | (1.01, 2.30) | 2.44 | (1.86, 3.44) | 1.18 | (0.89, 1.66) |
| C22:5 n-3 | 1.91 | (1.15, 2.85) | 3.33 | (2.32, 4.51) | 1.43 | (1.00, 2.06) |
| C22:6 n-3 | 35.56 | (24.98, 52.22) | 59.99 | (47.34, 74.40) | 28.51 | (21.99, 36.61) |
| N6/N3 ratio | 8.33 | (7.08, 9.94) | 8.83 | (7.57, 10.34) | 8.07 | (6.86, 9.48) |

SFA: saturated fatty acids; MUFA: monounsaturated fatty acids; PUFA: polyunsaturated fatty acids.

**Table S5.** Demographic characteristics of mother-infant dyads with different cord blood fatty acid concentrations.

|  | **Upper FA status** | | | **Lower FA status** | | *P* |
| --- | --- | --- | --- | --- | --- | --- |
|  | (n=197) | | | (n=393) | |  |
| ***Maternal characteristics*** | | | | | | |
| Maternal age, years | 31 | (29, 33) | | 30 | (28, 32) | 0.013 |
| Pre-pregnancy BMI | 21.8 | (19.9, 23.9) | | 21.5 | (19.9, 23.6) | 0.354 |
| Pre-pregnancy BMI category^a^ | | | |  |  | 0.064 |
| Underweight | 14 | 7.1 | | 51 | 13.0 |  |
| Normal weight | 149 | 75.6 | | 287 | 73.0 |  |
| Overweight | 31 | 15.7 | | 44 | 11.2 |  |
| Obese | 3 | 1.5 | | 11 | 2.8 |  |
| GWG | 15.0 | (11, 18) | | 15.0 | (11.9, 17.8) | 0.838 |
| Adequacy of GWG^b^ | | |  |  |  | 0.522 |
| Inadequate | 41 | 20.8 | | 75 | 19.1 |  |
| Adequate | 79 | 40.1 | | 177 | 45.0 |  |
| Excessive | 77 | 39.1 | | 141 | 35.9 |  |
| Primiparous | 86 | 43.7 | | 212 | 53.9 | 0.023 |
| Maternal birth weight category | | | |  |  | 0.415 |
| <2500g | 9 | 4.6 | | 14 | 3.6 |  |
| >=2500g, <4000g | 160 | 81.2 | | 324 | 82.4 |  |
| >=4000g | 8 | 4.1 | | 26 | 6.6 |  |
| Educational Level | | | |  |  | 0.425 |
| Secondary school or below | 48 | 24.4 | | 87 | 22.1 |  |
| College | 71 | 36.0 | | 129 | 32.8 |  |
| Undergraduate | 70 | 35.5 | | 149 | 37.9 |  |
| Postgraduate | 7 | 3.6 | | 25 | 6.4 |  |
| ***Infant characteristics*** | | | | | | |
| Sex, Male | 104 | 52.8 | | 221 | 56.2 | 0.481 |
| Gestational age, weeks | 39.0 | (38, 40) | | 39 | (38, 39) | 0.298 |
| Weight at birth, g | 3480 | (3200, 3750) | | 3490 | (3180, 3750) | 0.640 |
| Weight Z-Score^c^ | 0.8 | (0.2, 1.4) | | 0.79 | (0.16, 1.39) | 0.498 |
| Length at birth, cm | 50 | (49, 51) | | 50 | (49, 51) | 0.668 |
| Length Z-Score^c^ | 0.9 | (0.2, 1.4) | | 0.86 | (0.24, 1.37) | 0.696 |
| Weight-for-Length Ratio | 6.9 | (6.5, 7.4) | | 6.9 | (6.4, 7.4) | 0.718 |
| Weight-for-Length Z-Score^c^ | 0.5 | (-0.2, 1.1) | | 0.4 | (-0.29, 1.04) | 0.515 |

Abbreviations: BMI: body mass index; GWG: gestational weight gain: GHD: gestational hypertensive disorder; GDM: gestational diabetes mellitus.

^a^Definition which was based on the following standards: underweight (<18.5 kg/m^2^); normal weight (≥18.5, <25 kg/m^2^); overweight (≥25, <30 kg/m^2^) and obese (≥30 kg/m^2^).

^b^Adopted from the Institute of Medicine (IOM) Weight Gain Recommendation for Pregnancy (2009), adequate range of gestational weight gain for underweight, normal weight, overweight and obese mothers prior to pregnancy are 12.5-18kg, 11.5-16kg, 7-11.5kg and 5-9kg (inclusive), respectively. Weight gain below or above the recommended range were considered inadequate or excessive, respectively.

^c^Derived from INTERGROWTH-21^st^ standard.

Statistical comparisons between the two groups were performed with Kruskal-Wallis and Chi-squared test for continuous and categorical variables, respectively.

**Table S6.** Comparison of maternal serum PFASs level (ng/mL) in neonates of higher versus lower cord blood total fatty acids status.

| **Exposure** | **Upper FA status group** | | | | | | **Lower FA status group** | | | | | | ***P*** |
| --- | --- | --- | --- | --- | --- | --- | --- | --- | --- | --- | --- | --- | --- |
|  | **Min** | **1st Qu** | **Median** | **Mean** | **3rd Qu** | **Max.** | **Min** | **1st Qu** | **Median** | **Mean** | **3rd Qu** | **Max.** |  |
| Total PFASs | 8.23 | 15.0 | 18.0 | 20.2 | 24.6 | 57.2 | 6.91 | 14.9 | 20.0 | 21.1 | 25.8 | 52.3 | 0.190 |
| PFOA | 1.196 | 2.661 | 3.496 | 3.799 | 4.706 | 14.986 | 0.726 | 3.006 | 4.383 | 5.178 | 6.270 | 17.396 | **<0.001** |
| PFNA | 0.220 | 0.633 | 0.979 | 1.163 | 1.414 | 4.828 | 0.098 | 0.535 | 0.990 | 1.234 | 1.655 | 5.487 | 0.828 |
| PFOS | 1.320 | 4.082 | 6.220 | 7.066 | 8.830 | 23.578 | 0.396 | 2.970 | 5.130 | 6.004 | 7.388 | 28.190 | **<0.001** |
| PFDA | 0.106 | 0.582 | 0.952 | 1.306 | 1.728 | 5.936 | 0.126 | 0.566 | 0.948 | 1.177 | 1.545 | 7.146 | 0.367 |
| 6:2 Cl-PFESA | 0.200 | 0.591 | 1.050 | 1.270 | 1.680 | 7.013 | 0.118 | 0.761 | 1.231 | 1.548 | 1.983 | 7.560 | **0.005** |
| PFBA | 0.130 | 0.274 | 0.454 | 0.711 | 1.015 | 1.930 | <LOD | 0.322 | 0.573 | 0.732 | 1.056 | 2.496 | 0.183 |
| PFPeA | <LOD | 0.330 | 0.592 | 0.749 | 1.149 | 1.904 | 0.105 | 0.355 | 0.593 | 0.728 | 1.046 | 2.030 | 0.958 |
| PFUnDA | <LOD | 0.434 | 0.872 | 1.025 | 1.404 | 5.008 | 0.097 | 0.425 | 0.813 | 1.018 | 1.310 | 6.239 | 0.561 |
| PFHxA | <LOD | 0.115 | 0.171 | 0.248 | 0.258 | 1.718 | <LOD | 0.163 | 0.341 | 0.488 | 0.671 | 3.046 | **<0.001** |
| PFHxS | <LOD | 0.710 | 1.021 | 1.086 | 1.365 | 3.651 | <LOD | 0.535 | 1.055 | 1.223 | 1.694 | 5.836 | 0.508 |
| ADONA | 0.001 | 0.005 | 0.007 | 0.011 | 0.013 | 0.062 | 0.001 | 0.006 | 0.015 | 0.023 | 0.030 | 0.241 | **<0.001** |
| HFPO-DA | <LOD | 0.262 | 0.458 | 0.652 | 0.922 | 3.029 | <LOD | 0.179 | 0.387 | 0.591 | 0.897 | 2.885 | **0.014** |
| PFTrDA | <LOD | 0.068 | 0.117 | 0.149 | 0.195 | 0.667 | <LOD | 0.057 | 0.111 | 0.145 | 0.192 | 0.944 | 0.335 |
| PFHpA | <LOD | 0.040 | 0.074 | 0.099 | 0.129 | 0.372 | <LOD | 0.041 | 0.112 | 0.190 | 0.227 | 2.555 | **0.001** |
| 6:2FTS | <LOD | 0.070 | 0.119 | 0.147 | 0.187 | 0.739 | <LOD | <LOD | 0.072 | 0.114 | 0.163 | 0.869 | **<0.001** |
| PFDoDA | <LOD | 0.072 | 0.117 | 0.176 | 0.206 | 1.414 | <LOD | <LOD | 0.082 | 0.125 | 0.166 | 1.064 | **<0.001** |

**Table S7.** Posterior Inclusion Probability of sixteen PFASs included in the Bayesian Kernal Machine Regression modelling for analysing the PFASs mixture effect on newborn size parameters, adjusted for maternal age, maternal birthweight category (<2500g, ≥2500g & <4000g, ≥4000g), maternal pre-pregnancy body mass index, gestational weight gain, sex of newborn, gestational age at birth.

|  | **Total population** | | | **Upper FA status** | | | **Lower FA status** | | |
| --- | --- | --- | --- | --- | --- | --- | --- | --- | --- |
|  | **WZ** | **LZ** | **WLRZ** | **WZ** | **LZ** | **WLRZ** | **WZ** | **LZ** | **WLRZ** |
| PFBA | 0.503 | 0.072 | 0.606 | 0.256 | 0.194 | 0.189 | 0.808 | 0.279 | 0.663 |
| PFPeA | 0.266 | 0.085 | 0.604 | 0.254 | 0.182 | 0.193 | 0.486 | 0.305 | 0.129 |
| PFHxA | 0.374 | 0.145 | 0.614 | 0.314 | 0.199 | 0.296 | 0.309 | 0.372 | 0.028 |
| HFPO-DA | 0.510 | 0.080 | 0.772 | 0.308 | 0.175 | 0.288 | 0.408 | 0.233 | 0.046 |
| PFHpA | 0.156 | 0.062 | 0.503 | 0.262 | 0.216 | 0.206 | 0.278 | 0.233 | 0.024 |
| ADONA | 0.142 | 0.070 | 0.561 | 0.385 | 0.203 | 0.312 | 0.239 | 0.214 | 0.010 |
| PFHxS | 0.166 | 0.090 | 0.554 | 0.372 | 0.257 | 0.282 | 0.282 | 0.209 | 0.020 |
| PFOA | 0.710 | 0.276 | 0.751 | 0.353 | 0.196 | 0.329 | 0.474 | 0.380 | 0.088 |
| 6: 2FTS | 0.206 | 0.077 | 0.513 | 0.224 | 0.221 | 0.224 | 0.340 | 0.258 | 0.040 |
| PFNA | 0.255 | 0.073 | 0.649 | 0.293 | 0.313 | 0.239 | 0.363 | 0.235 | 0.110 |
| PFOS | 0.210 | 0.121 | 0.622 | 0.210 | 0.289 | 0.205 | 0.271 | 0.231 | 0.013 |
| PFDA | 0.280 | 0.099 | 0.624 | 0.231 | 0.225 | 0.245 | 0.363 | 0.249 | 0.036 |
| 6:2 Cl-PFESA | 0.311 | 0.122 | 0.619 | 0.269 | 0.295 | 0.233 | 0.326 | 0.259 | 0.068 |
| PFUnDA | 0.162 | 0.051 | 0.580 | 0.221 | 0.203 | 0.213 | 0.259 | 0.282 | 0.009 |
| PFDoDA | 0.257 | 0.146 | 0.576 | 0.698 | 0.954 | 0.362 | 0.335 | 0.254 | 0.057 |
| PFTrDA | 0.175 | 0.076 | 0.581 | 0.208 | 0.215 | 0.245 | 0.349 | 0.254 | 0.030 |

Abbreviations: LZ: length Z-score; WZ: weight Z-score; WLRZ: weight-for-length ratio Z-score.

**Table S8.** Mixture of all PFASs with detection frequency of greater than 60% (n=16) on cord blood fatty acid level as determined by quantile-based g-computation model, weight of individual exposures only shown for those significantly affected fatty acids. Corresponding to Figure 3.

|  | **Total FAs** | **Total SFA** | **C14:0** | **C15:0** | **C16:0** | **C17:0** | **C18:0** | **C19:0** | **C20:0** | **C22:0** |
| --- | --- | --- | --- | --- | --- | --- | --- | --- | --- | --- |
| **β** | -0.14 | -0.15 | -0.13 | -0.13 | -0.16 | -0.12 | -0.13 | -0.26 | -0.25 | -0.23 |
| **95% CI** | (-0.25,  -0.03) | (-0.26,  -0.04) | (-0.25,  -0.01) | (-0.25,  -0.02) | (-0.28,  -0.04) | (-0.22,  -0.01) | (-0.24,  -0.02) | (-0.45,  -0.08) | (-0.39,  -0.12) | (-0.35,  -0.12) |
| ***Weight carried by individual exposures*** | | | | | | | | | | |
| PFOA | -0.149 | -0.151 | -0.153 | -0.160 | -0.142 | -0.134 | -0.163 | -0.157 | -0.120 | -0.151 |
| PFNA | -0.039 | -0.046 | -0.039 | -0.044 | -0.035 | -0.053 | -0.057 | -0.100 | -0.223 | -0.088 |
| PFOS | 0.288 | 0.277 | 0.297 | 0.278 | 0.273 | 0.310 | 0.263 | 0.255 | 0.457 | 0.369 |
| PFDA | 0.001 | -0.007 | -0.006 | -0.008 | -0.010 | 0.026 | -0.013 | -0.003 | -0.006 | 0.066 |
| 6:2 Cl-PFESA | -0.181 | -0.178 | -0.207 | -0.219 | -0.181 | -0.190 | -0.167 | -0.137 | 0.011 | -0.145 |
| PFBA | -0.037 | -0.037 | -0.024 | -0.004 | -0.038 | -0.002 | -0.027 | -0.029 | -0.001 | -0.079 |
| PFPeA | 0.007 | 0.000 | -0.015 | -0.003 | -0.009 | -0.013 | 0.027 | -0.025 | -0.016 | -0.004 |
| PFUnDA | -0.002 | 0.006 | 0.004 | 0.000 | -0.007 | 0.018 | 0.047 | 0.072 | -0.037 | 0.001 |
| PFHxA | -0.278 | -0.277 | -0.242 | -0.229 | -0.268 | -0.293 | -0.294 | -0.311 | -0.236 | -0.225 |
| PFHxS | -0.009 | -0.022 | -0.008 | -0.005 | -0.024 | 0.002 | -0.020 | 0.017 | -0.009 | -0.049 |
| ADONA | -0.211 | -0.197 | -0.215 | -0.218 | -0.201 | -0.211 | -0.180 | -0.149 | -0.231 | -0.176 |
| HFPO-DA | 0.120 | 0.101 | 0.142 | 0.114 | 0.108 | 0.020 | 0.074 | 0.106 | 0.087 | 0.093 |
| PFTrDA | 0.119 | 0.137 | 0.100 | 0.134 | 0.150 | 0.135 | 0.110 | 0.134 | 0.144 | 0.085 |
| PFHpA | -0.094 | -0.085 | -0.092 | -0.112 | -0.085 | -0.104 | -0.079 | -0.090 | -0.117 | -0.082 |
| 6: 2FTS | 0.236 | 0.242 | 0.199 | 0.181 | 0.220 | 0.289 | 0.278 | 0.237 | 0.301 | 0.238 |
| PFDoDA | 0.229 | 0.237 | 0.257 | 0.294 | 0.249 | 0.201 | 0.201 | 0.178 | -0.005 | 0.148 |
|  | **C24:0** | **Total MUFA** | **C16:1**  **n-7** | **C18:1**  **n-9** | **C18:1**  **n-7** | **C20:1**  **n-9** | **C18:2**  **n-6** | **C20:2**  **n-6** | **C20:3**  **n-6** | **C22:5**  **n-3** |
| **β** | -0.27 | -0.18 | -0.15 | -0.19 | -0.21 | -0.14 | -0.15 | -0.19 | -0.14 | -0.21 |
| **95% CI** | (-0.47,  -0.07) | (-0.30,  -0.06) | (-0.26,  -0.03) | (-0.31,  -0.06) | (-0.36,  -0.06) | (-0.25,  -0.03) | (-0.27,  -0.03) | (-0.30,  -0.08) | (-0.26,  -0.02) | (-0.38,  -0.04) |
| ***Weight carried by individual exposures*** | | | | | | | | | | |
| PFOA | -0.049 | -0.142 | -0.114 | -0.141 | -0.128 | -0.061 | -0.138 | -0.133 | -0.218 | -0.194 |
| PFNA | 0.290 | -0.001 | -0.022 | -0.010 | 0.087 | -0.059 | -0.061 | -0.039 | 0.065 | 0.052 |
| PFOS | -0.001 | 0.316 | 0.290 | 0.319 | 0.236 | 0.374 | 0.268 | 0.472 | 0.257 | 0.223 |
| PFDA | -0.097 | 0.054 | 0.041 | 0.054 | 0.095 | 0.057 | -0.016 | -0.024 | -0.023 | -0.044 |
| 6:2 Cl-PFESA | -0.005 | -0.178 | -0.149 | -0.180 | -0.161 | -0.208 | -0.190 | -0.170 | -0.167 | -0.197 |
| PFBA | -0.243 | -0.021 | -0.022 | -0.016 | -0.078 | 0.032 | -0.039 | -0.058 | -0.076 | -0.132 |
| PFPeA | -0.088 | -0.006 | -0.026 | -0.007 | -0.001 | 0.005 | -0.018 | 0.002 | -0.011 | 0.029 |
| PFUnDA | 0.094 | -0.008 | -0.044 | -0.001 | -0.020 | -0.035 | 0.020 | -0.038 | 0.051 | 0.030 |
| PFHxA | -0.243 | -0.292 | -0.282 | -0.290 | -0.259 | -0.204 | -0.234 | -0.216 | -0.229 | -0.165 |
| PFHxS | 0.248 | -0.021 | -0.036 | -0.028 | 0.111 | -0.049 | 0.013 | -0.048 | -0.007 | 0.050 |
| ADONA | -0.246 | -0.221 | -0.231 | -0.210 | -0.303 | -0.217 | -0.220 | -0.199 | -0.201 | -0.135 |
| HFPO-DA | 0.248 | 0.124 | 0.107 | 0.110 | 0.275 | 0.071 | 0.134 | 0.037 | 0.039 | 0.079 |
| PFTrDA | 0.039 | 0.080 | 0.068 | 0.083 | 0.130 | -0.027 | 0.105 | 0.104 | 0.128 | 0.211 |
| PFHpA | 0.025 | -0.110 | -0.073 | -0.118 | -0.050 | -0.139 | -0.084 | -0.076 | -0.066 | -0.133 |
| 6: 2FTS | -0.028 | 0.241 | 0.304 | 0.241 | 0.044 | 0.248 | 0.235 | 0.227 | 0.242 | 0.059 |
| PFDoDA | 0.056 | 0.184 | 0.190 | 0.193 | 0.023 | 0.213 | 0.226 | 0.158 | 0.218 | 0.268 |

Abbreviations: CI: confidence interval; FA: fatty acids; MUFA: monounsaturated fatty acids; SFA: saturated fatty acids.

**Table S9.** Association between selected cord blood fatty acids (log transformed) and newborn size parameters determined by multivariate linear model, adjusted for maternal age, maternal birthweight category (<2500g, ≥2500g & <4000g, ≥4000g), maternal pre-pregnancy body mass index, gestational weight gain, sex of newborn, gestational age at birth.

|  | **Weight Z-Score** | | | **Length Z-Score** | | | **Weight-for-Length Ratio Z-Score** | | |
| --- | --- | --- | --- | --- | --- | --- | --- | --- | --- |
|  | Beta | 95% CI | *P* | Beta | 95% CI | *P* | Beta | 95% CI | *P* |
| Total SFA | 0.020 | (-0.103, 0.143) | 0.748 | 0.046 | (-0.093, 0.185) | 0.515 | 0.027 | (-0.110, 0.163) | 0.701 |
| C14:0 | -0.024 | (-0.139, 0.090) | 0.678 | 0.029 | (-0.100, 0.158) | 0.660 | -0.019 | (-0.146, 0.107) | 0.763 |
| C15:0 | -0.010 | (-0.132, 0.111) | 0.867 | 0.058 | (-0.079, 0.195) | 0.408 | -0.004 | (-0.139, 0.130) | 0.948 |
| C16:0 | 0.020 | (-0.100, 0.139) | 0.749 | 0.055 | (-0.079, 0.190) | 0.421 | 0.022 | (-0.110, 0.155) | 0.741 |
| C17:0 | -0.005 | (-0.140, 0.129) | 0.938 | 0.039 | (-0.112, 0.191) | 0.611 | 0.007 | (-0.142, 0.156) | 0.929 |
| C18:0 | 0.022 | (-0.103, 0.147) | 0.734 | 0.023 | (-0.118, 0.164) | 0.753 | 0.035 | (-0.103, 0.173) | 0.619 |
| C19:0 | 0.031 | (-0.041, 0.103) | 0.397 | 0.007 | (-0.074, 0.088) | 0.860 | 0.047 | (-0.033, 0.126) | 0.249 |
| C20:0 | 0.068 | (-0.035, 0.171) | 0.196 | 0.023 | (-0.093, 0.140) | 0.695 | 0.091 | (-0.023, 0.205) | 0.120 |
| C22:0 | 0.121 | (0.002, 0.240) | 0.047 | 0.093 | (-0.041, 0.228) | 0.175 | 0.135 | (0.003, 0.267) | 0.045 |
| C24:0 | -0.012 | (-0.085, 0.061) | 0.749 | -0.023 | (-0.106, 0.060) | 0.589 | -0.003 | (-0.084, 0.078) | 0.942 |
| Total MUFA | 0.008 | (-0.106, 0.122) | 0.888 | 0.019 | (-0.110, 0.147) | 0.777 | 0.020 | (-0.106, 0.146) | 0.757 |
| C16:1 n-7 | 0.075 | (-0.046, 0.197) | 0.224 | 0.063 | (-0.075, 0.200) | 0.372 | 0.086 | (-0.049, 0.220) | 0.213 |
| C18:1 n-9 | 0.000 | (-0.109, 0.109) | 0.994 | 0.011 | (-0.112, 0.134) | 0.857 | 0.012 | (-0.109, 0.132) | 0.850 |
| C18:1 n-7 | 0.012 | (-0.083, 0.107) | 0.809 | 0.054 | (-0.054, 0.161) | 0.326 | 0.006 | (-0.099, 0.112) | 0.907 |
| C18:2 n-6 | -0.039 | (-0.154, 0.077) | 0.514 | -0.018 | (-0.148, 0.113) | 0.792 | -0.016 | (-0.144, 0.112) | 0.807 |
| C20:2 n-6 | 0.183 | (0.053, 0.312) | 0.006 | 0.118 | (-0.029, 0.264) | 0.116 | 0.194 | (0.051, 0.337) | 0.008 |
| C20:3 n-6 | 0.068 | (-0.046, 0.182) | 0.245 | 0.060 | (-0.068, 0.189) | 0.360 | 0.079 | (-0.048, 0.205) | 0.223 |
| C22:5 n-3 | 0.071 | (-0.016, 0.159) | 0.109 | 0.078 | (-0.020, 0.176) | 0.120 | 0.083 | (-0.014, 0.179) | 0.094 |

*P* value indicates whether there is statistically significant association between cord blood fatty acids and newborn size.

**Table S10.** Association between selected PFASs and newborn size parameters were modified by selected fatty acids (upper versus lower), coefficient and 95% confidence interval along with *P* values were reported for multivariate linear models, while only *P* values were reported for restricted cubic spline model. All models were adjusted for maternal age, maternal birthweight category (<2500g, ≥2500g & <4000g, ≥4000g), maternal pre-pregnancy body mass index, gestational weight gain, sex of newborn, gestational age at birth.

| Fatty acids Status | | **PFBA** | | | | | |
| --- | --- | --- | --- | --- | --- | --- | --- |
|  |  | **Weight Z-score** | | | **Weight-for-Length Ratio Z-score** | | |
|  |  | **β** | **95% CI** | ***P*** | **β** | **95% CI** | ***P*** |
| Total SFA | Lower | -0.19 | (-0.30, -0.08) | 0.001 | -0.21 | (-0.33, -0.08) | 0.001 |
|  | Upper | -0.01 | (-0.16, 0.13) | 0.846 | -0.00 | (-0.15, 0.15) | 0.994 |
| C16:0 | Lower | -0.19 | (-0.30, -0.08) | <0.001 | -0.21 | (-0.33, -0.08) | 0.001 |
|  | Upper | -0.01 | (-0.15, 0.13) | 0.896 | -0.00 | (-0.16, 0.15) | 0.960 |
| C18:0 | Lower | Non-significant interaction | | | -0.21 | (-0.33, -0.08) | 0.001 |
|  | Upper |  |  |  | 0.00 | (-0.15, 0.15) | 0.988 |
| C18:2 n-6 | Lower | Non-significant interaction | | | -0.20 | (-0.33, -0.08) | 0.001 |
|  | Upper |  |  |  | -0.04 | (-0.19, 0.12) | 0.655 |
|  |  | **HFPO-DA** | | | | | |
| C20:1 n-9 | Lower | -0.10 | (-0.17, -0.03) | 0.005 | -0.11 | (-0.19, -0.03) | 0.006 |
|  | Upper | 0.05 | (-0.07, 0.17) | 0.394 | 0.08 | (-0.05, 0.21) | 0.201 |
|  |  | **PFPeA** | | | | | |
| C14:0 | Lower | -0.19 | (-0.31, -0.06) | 0.003 | -0.19 | (-0.33, -0.05) | 0.007 |
|  | Upper |  |  | 0.030 |  |  | 0.020 |
| C16:0 | Lower | -0.14 | (-0.26, -0.02) | 0.026 | -0.16 | (-0.29, -0.02) | 0.023 |
|  | Upper |  |  | 0.004 |  |  | 0.002 |
| C18:0 | Lower | Non-significant interaction | | | -0.15 | (-0.29, -0.01) | 0.033 |
|  | Upper |  |  |  |  |  | <0.001 |
| C16:1 n-7 | Lower | -0.15 | (-0.27, -0.03) | 0.018 | -0.17 | (-0.30, -0.03) | 0.015 |
|  | Upper |  |  | 0.003 |  |  | 0.002 |

**Table S11.** Significant level of the effect modification by cord blood fatty acids on the association between individual PFASs exposure and newborn size parameters, determined by generalized linear regression or restricted cubic spline regression. All models were adjusted for maternal age, maternal birthweight category (<2500g, ≥2500g & <4000g, ≥4000g), maternal pre-pregnancy body mass index, gestational weight gain, sex of newborn, gestational age at birth.

| **Moderator** | **Weight Z-Score** | | | | | | **Weight-for-Length Ratio Z-score** | | | | | | **Length Z-Score** | |
| --- | --- | --- | --- | --- | --- | --- | --- | --- | --- | --- | --- | --- | --- | --- |
|  | **LR** | | | **RCS** | | | **LR** | | | **RCS** | | | **LR** | **RCS** |
|  | **PFBA** | **HFPO-DA** | **PFOA** | **PFPeA** | **PFHxA** | **PFNA** | **PFBA** | **HFPO-DA** | **PFOA** | **PFPeA** | **PFHxA** | **PFNA** | **PFOA** | **6:2FTS** |
| Total SFA | **0.043** | 0.471 | 0.539 | 0.191 | 0.649 | 0.536 | **0.031** | 0.381 | 0.499 | 0.051 | 0.681 | 0.570 | 0.839 | 0.289 |
| C14:0 | 0.076 | 0.818 | 0.321 | **0.009** | 0.881 | 0.330 | 0.116 | 0.614 | 0.250 | **0.023** | 0.956 | 0.266 | 0.991 | 0.248 |
| C15:0 | 0.341 | 0.489 | 0.938 | 0.070 | 0.773 | 0.176 | 0.417 | 0.590 | 0.769 | 0.060 | 0.896 | 0.205 | 0.328 | 0.233 |
| C16:0 | **0.032** | 0.548 | 0.521 | **0.041** | 0.705 | 0.614 | **0.031** | 0.415 | 0.505 | **0.010** | 0.765 | 0.653 | 0.832 | 0.372 |
| C17:0 | 0.135 | 0.644 | 0.386 | 0.391 | 0.259 | 0.181 | 0.103 | 0.707 | 0.508 | 0.251 | 0.345 | 0.149 | 0.225 | 0.099 |
| C18:0 | 0.071 | 0.258 | 0.571 | 0.121 | 0.502 | 0.556 | **0.025** | 0.144 | 0.821 | **0.041** | 0.630 | 0.537 | 0.394 | 0.245 |
| C19:0 | 0.460 | 0.754 | 0.190 | 0.086 | 0.098 | 0.824 | 0.836 | 0.399 | 0.191 | 0.313 | 0.118 | 0.600 | 0.434 | 0.239 |
| C20:0 | 0.133 | 0.967 | 0.190 | 0.752 | 0.397 | 0.671 | 0.156 | 0.790 | 0.179 | 0.985 | 0.320 | 0.748 | 0.407 | 0.733 |
| C22:0 | 0.240 | 0.341 | 0.166 | 0.874 | 0.342 | 0.649 | 0.252 | 0.433 | 0.057 | 0.911 | 0.394 | 0.575 | 0.965 | 0.515 |
| C24:0 | 0.128 | 0.853 | 0.271 | 0.593 | 0.481 | 0.542 | 0.097 | 0.811 | 0.160 | 0.792 | 0.429 | 0.415 | 0.978 | 0.770 |
| Total MUFA | 0.390 | 0.174 | 0.722 | 0.209 | 0.605 | 0.695 | 0.229 | 0.126 | 0.836 | 0.077 | 0.586 | 0.731 | 0.836 | 0.490 |
| C16:1 n-7 | 0.799 | 0.662 | 0.924 | **0.003** | 0.215 | 0.620 | 0.375 | 0.439 | 0.681 | **0.002** | 0.207 | 0.830 | 0.619 | 0.173 |
| C18:1 n-9 | 0.220 | 0.413 | 0.939 | 0.282 | 0.674 | 0.776 | 0.119 | 0.330 | 0.921 | 0.114 | 0.721 | 0.867 | 0.814 | 0.541 |
| C18:1 n-7 | 0.081 | 0.382 | 0.458 | 0.632 | 0.366 | 0.582 | 0.092 | 0.238 | 0.507 | 0.288 | 0.364 | 0.450 | 0.678 | 0.787 |
| C20:1 n-9 | 0.307 | **0.047** | 0.282 | 0.401 | 0.213 | 0.299 | 0.145 | **0.015** | 0.431 | 0.186 | 0.449 | 0.396 | 0.444 | 0.793 |
| C18:2 n-6 | 0.388 | 0.459 | 0.360 | 0.190 | 0.563 | 0.673 | **0.036** | 0.567 | 0.399 | 0.137 | 0.706 | 0.617 | 0.681 | 0.095 |
| C20:2 n-6 | 0.181 | 0.418 | 0.412 | 0.110 | 0.8756 | 0.706 | 0.134 | 0.336 | 0.542 | **0.017** | 0.697 | 0.879 | 0.315 | 0.444 |
| C20:3 n-6 | 0.315 | 0.157 | 0.511 | 0.303 | 0.351 | 0.987 | 0.401 | 0.159 | 0.627 | 0.167 | 0.274 | 0.915 | 0.423 | 0.394 |
| C22:5 n-3 | 0.578 | 0.800 | 0.952 | 0.740 | 0.144 | 0.706 | 0.505 | 0.735 | 0.959 | 0.485 | 0.126 | 0.958 | 0.657 | 0.234 |

LR: linear regression model; RCS: restricted cubic spline regression model.

**
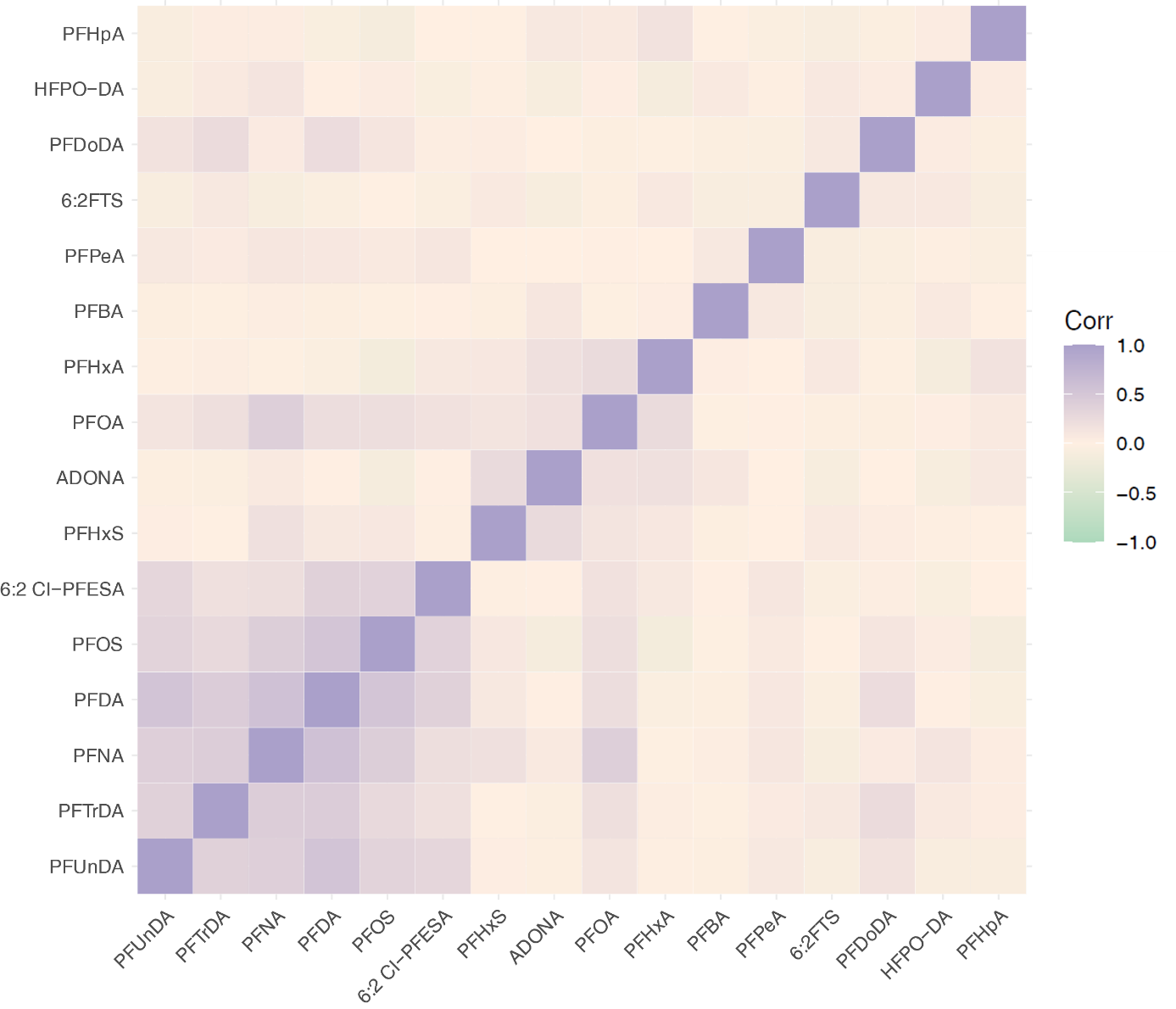
**

**Figure S1.** Spearman correlations between all 16 PFASs with detection frequency above 60%.


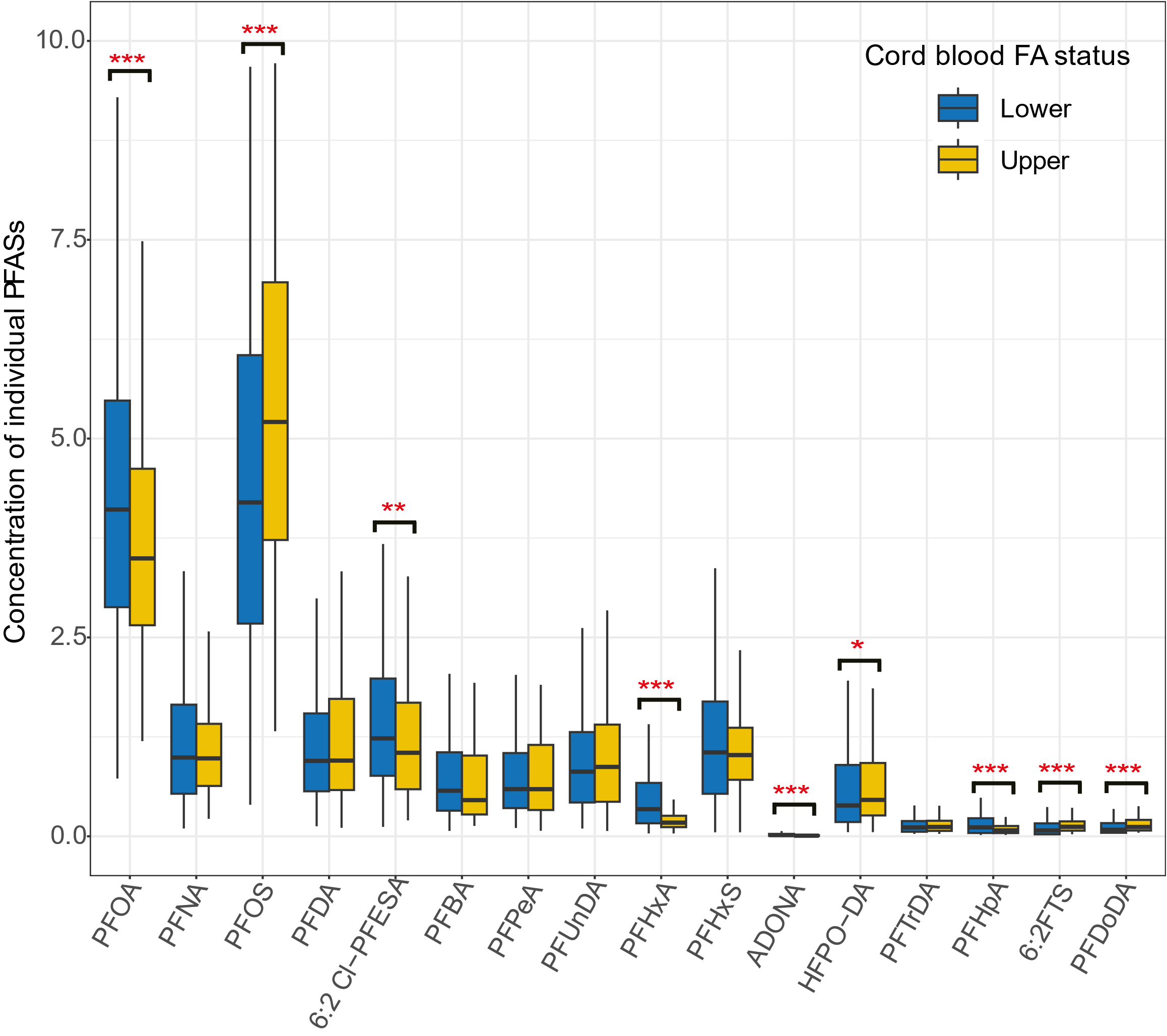


**Figure S2.** Comparison of maternal serum PFASs level (ng/mL) in neonates of higher versus lower cord blood total fatty acids level (μg/mL). Starts indicating significance level, where **P*<0.05, ***P*<0.01, ****P*<0.001.


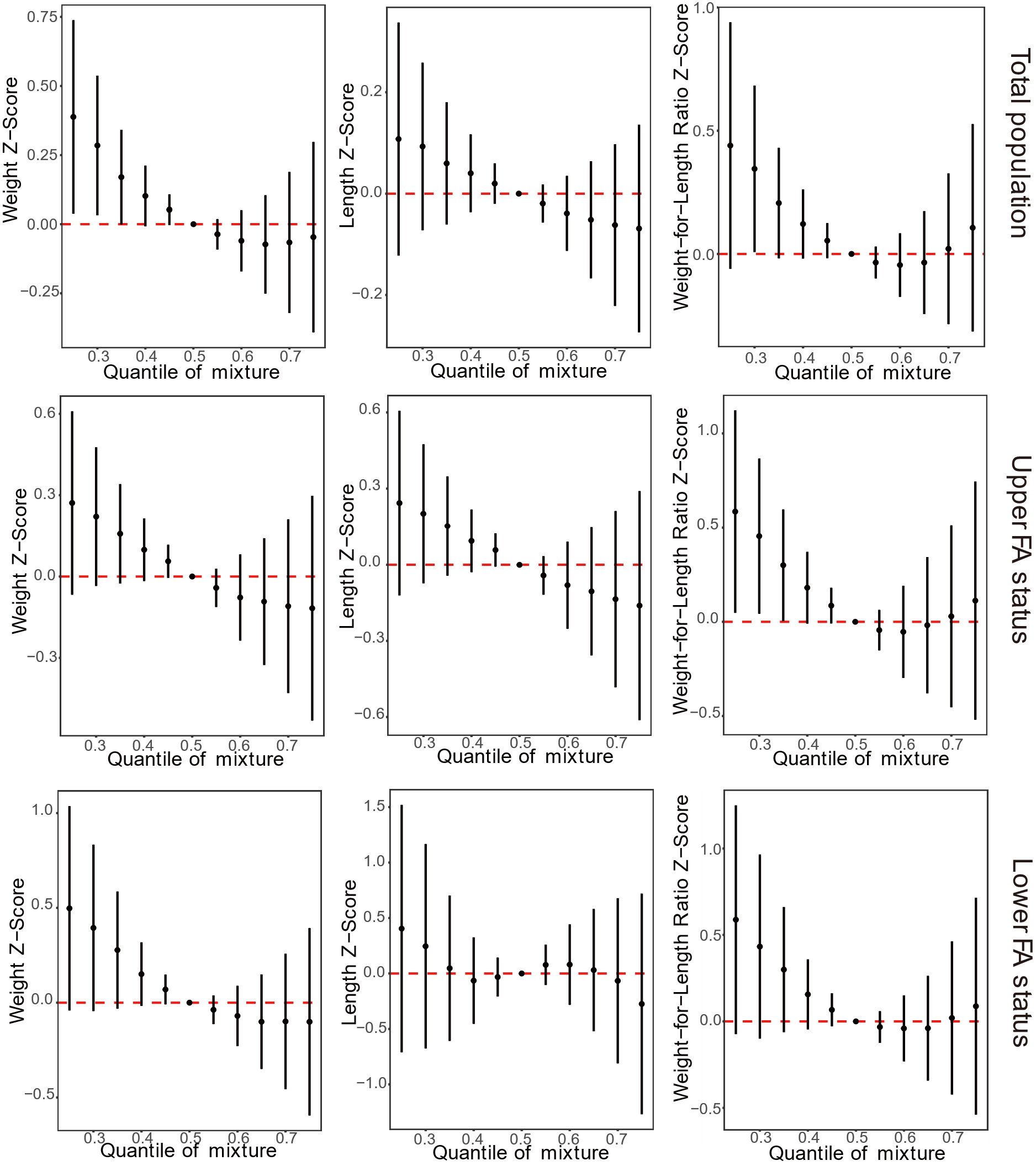


**Figure S3.** Mixture effect of PFASs (detection frequency ≥60%, n=16) on newborn size parameters (Weight Z-Score, Length Z-Score and Weight-for-Length Ratio Z-score) in the total population and the population stratified by cord blood fatty acid status, by Bayesian Kernal Regression modelling. The model was adjusted for maternal age, maternal birthweight category (<2500g, ≥2500g & <4000g, ≥4000g), maternal pre-pregnancy body mass index, gestational weight gain, sex of newborn, gestational age at birth.
